# Supplementary material for: Estimating total human deaths from rabies: A statistical framework leveraging reports of rabies transmission from deceased organ and tissue donors
Source: PLoS Negl Trop Dis. 2026 Sep 21;20(9):e0014731. doi: 10.1371/journal.pntd.0014731 (PMC13592719; doi:10.1371/journal.pntd.0014731)
Supplement: Supplementary file 6 [file pntd.0014731.s005.zip › Figure_Collection.pdf]

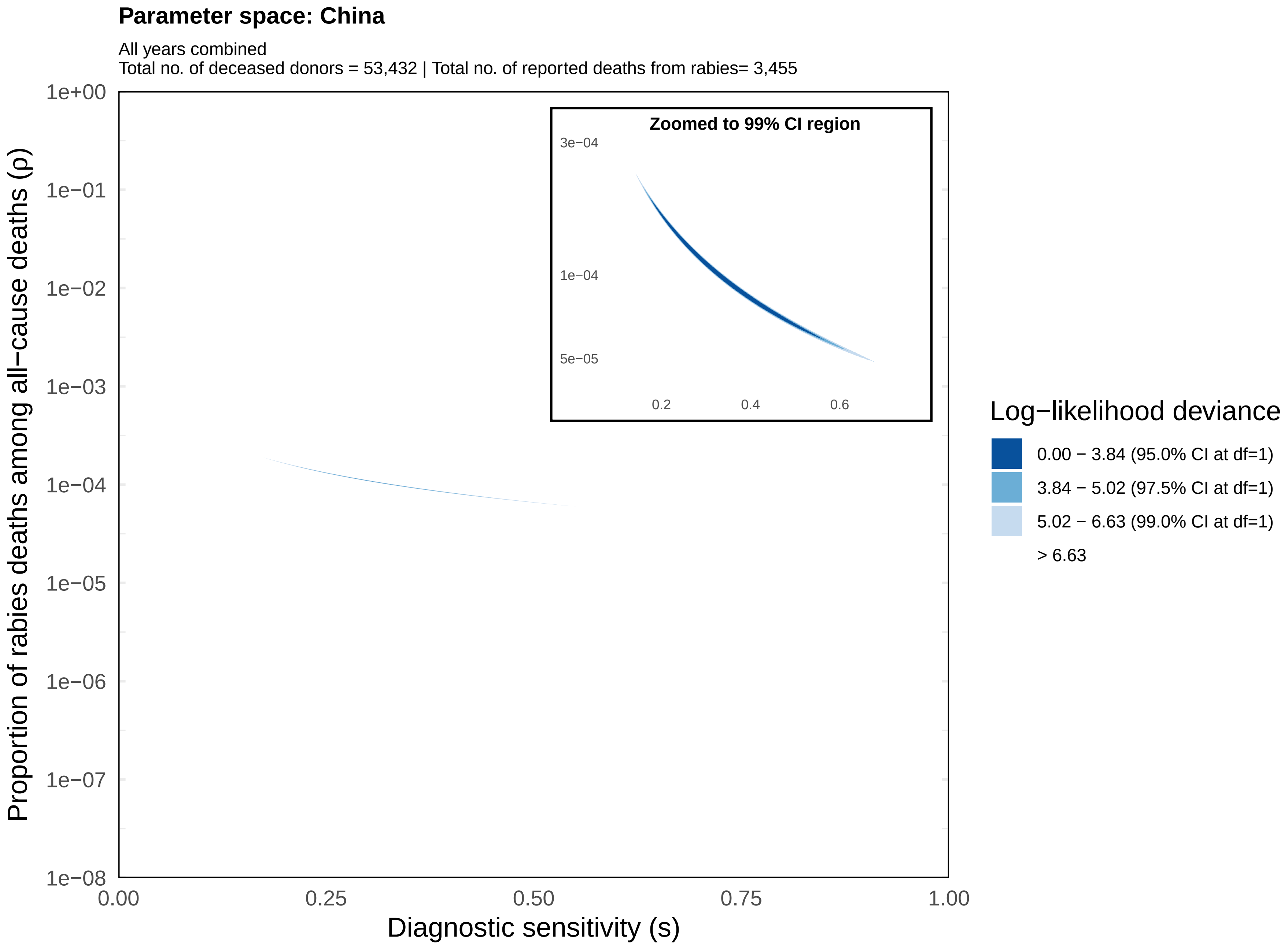

Parameter space: China
All years combined
Total no. of deceased donors = 53,432 | Total no. of reported deaths from rabies= 3,455
Proportion of rabies deaths among all−cause deaths (ρ)
1e+00
1e−01
1e−02
1e−03
1e−04
1e−05
1e−06
1e−07
1e−08
0.00
0.25
0.50
0.75
1.00
Diagnostic sensitivity (s)
Zoomed to 99% CI region
3e−04
1e−04
5e−05
0.2
0.4
0.6
Log−likelihood deviance
0.00 − 3.84 (95.0% CI at df=1)
3.84 − 5.02 (97.5% CI at df=1)
5.02 − 6.63 (99.0% CI at df=1)
> 6.63

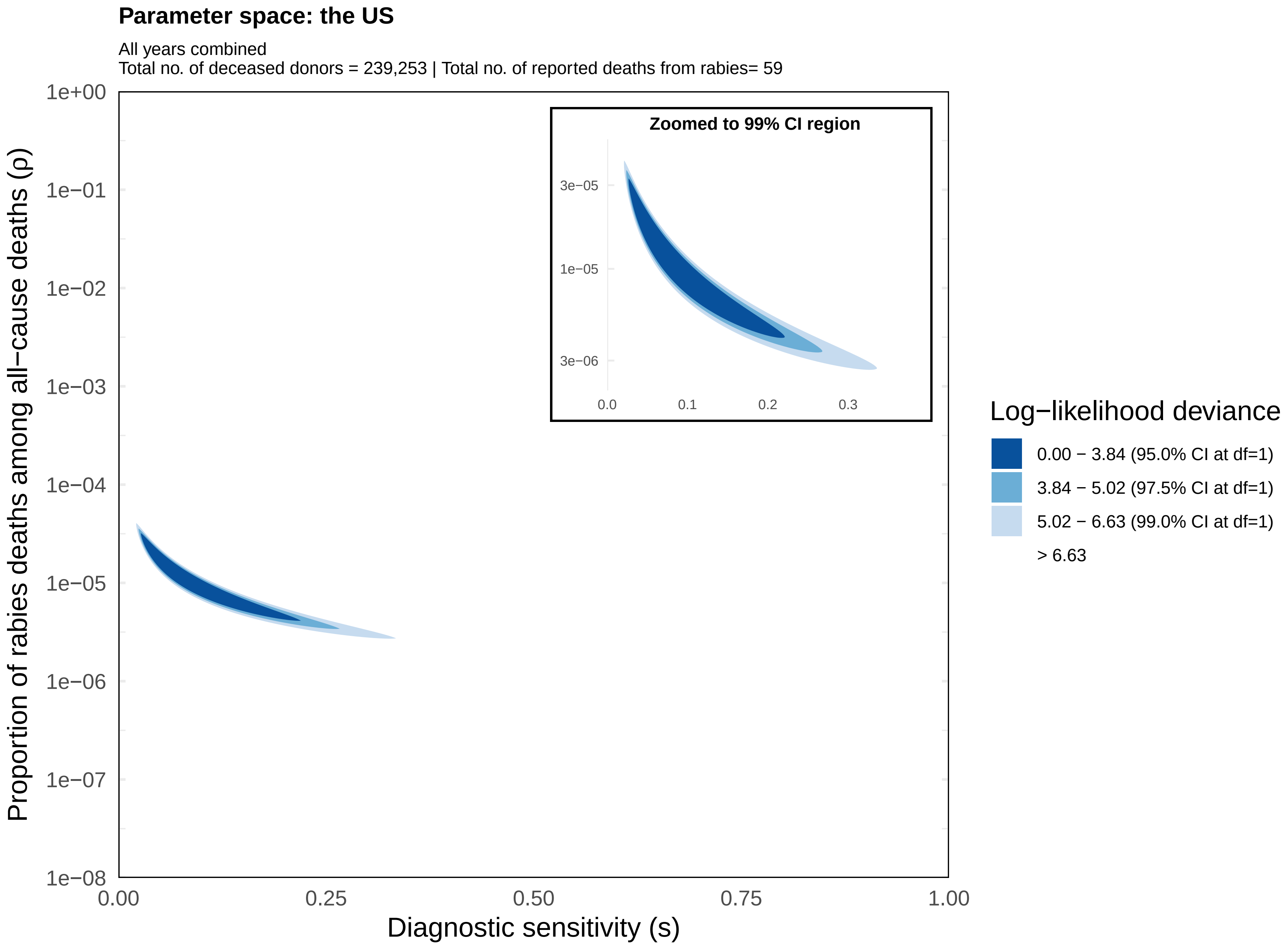

Parameter space: the US
All years combined
Total no. of deceased donors = 239,253 | Total no. of reported deaths from rabies= 59
Proportion of rabies deaths among all−cause deaths (ρ)
1e+00
1e−01
1e−02
1e−03
1e−04
1e−05
1e−06
1e−07
1e−08
0.00
0.25
0.50
0.75
1.00
Diagnostic sensitivity (s)
Zoomed to 99% CI region
3e−05
1e−05
3e−06
0.0
0.1
0.2
0.3
Log−likelihood deviance
0.00 − 3.84 (95.0% CI at df=1)
3.84 − 5.02 (97.5% CI at df=1)
5.02 − 6.63 (99.0% CI at df=1)
> 6.63

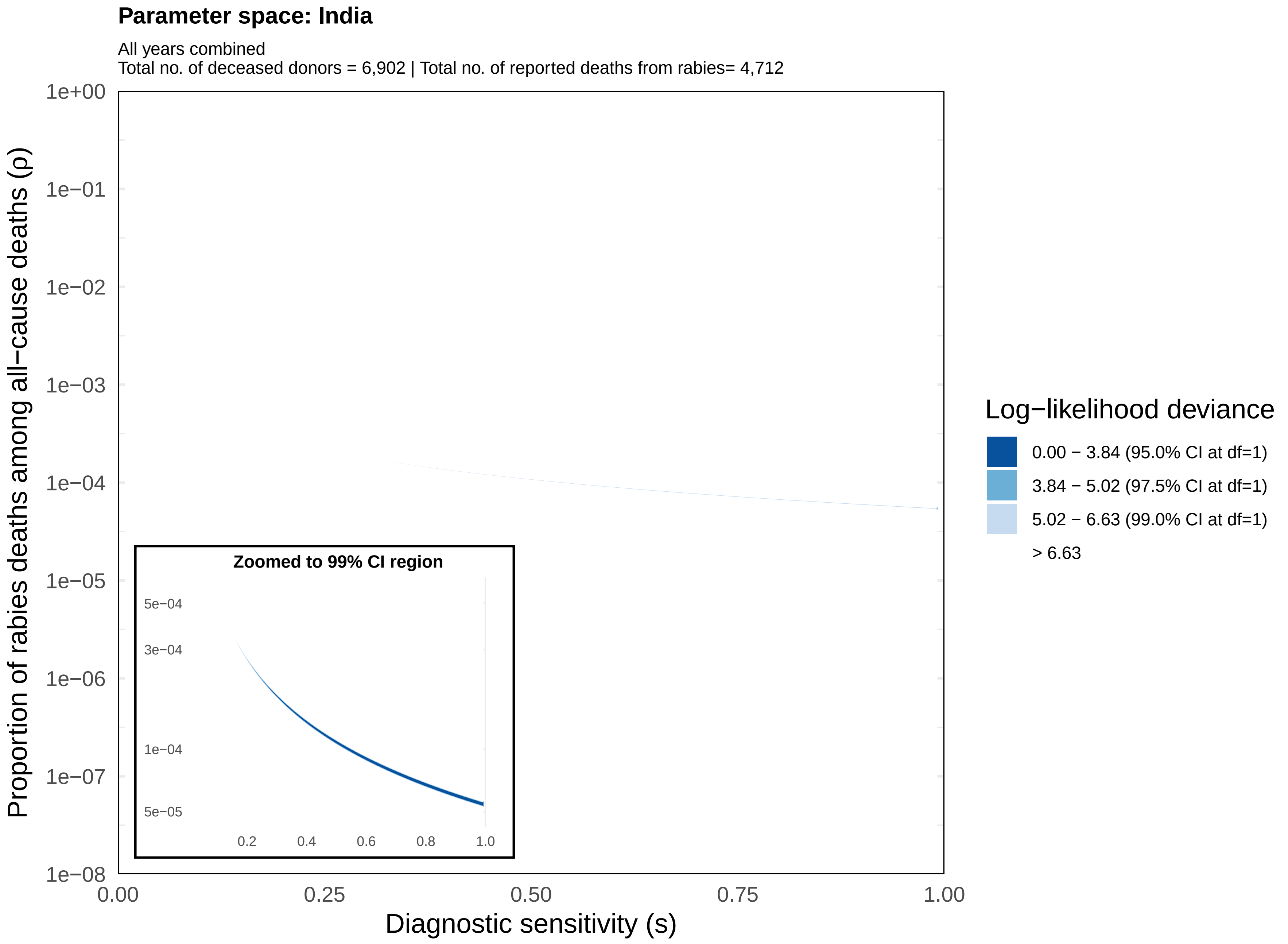

Parameter space: India
All years combined
Total no. of deceased donors = 6,902 | Total no. of reported deaths from rabies= 4,712
1e+00
1e−01
1e−02
1e−03
1e−04
1e−05
1e−06
1e−07
1e−08
Proportion of rabies deaths among all−cause deaths (ρ)
0.00
0.25
0.50
0.75
1.00
Diagnostic sensitivity (s)
Zoomed to 99% CI region
5e−04
3e−04
1e−04
5e−05
0.2
0.4
0.6
0.8
1.0
Log−likelihood deviance
0.00 − 3.84 (95.0% CI at df=1)
3.84 − 5.02 (97.5% CI at df=1)
5.02 − 6.63 (99.0% CI at df=1)
> 6.63

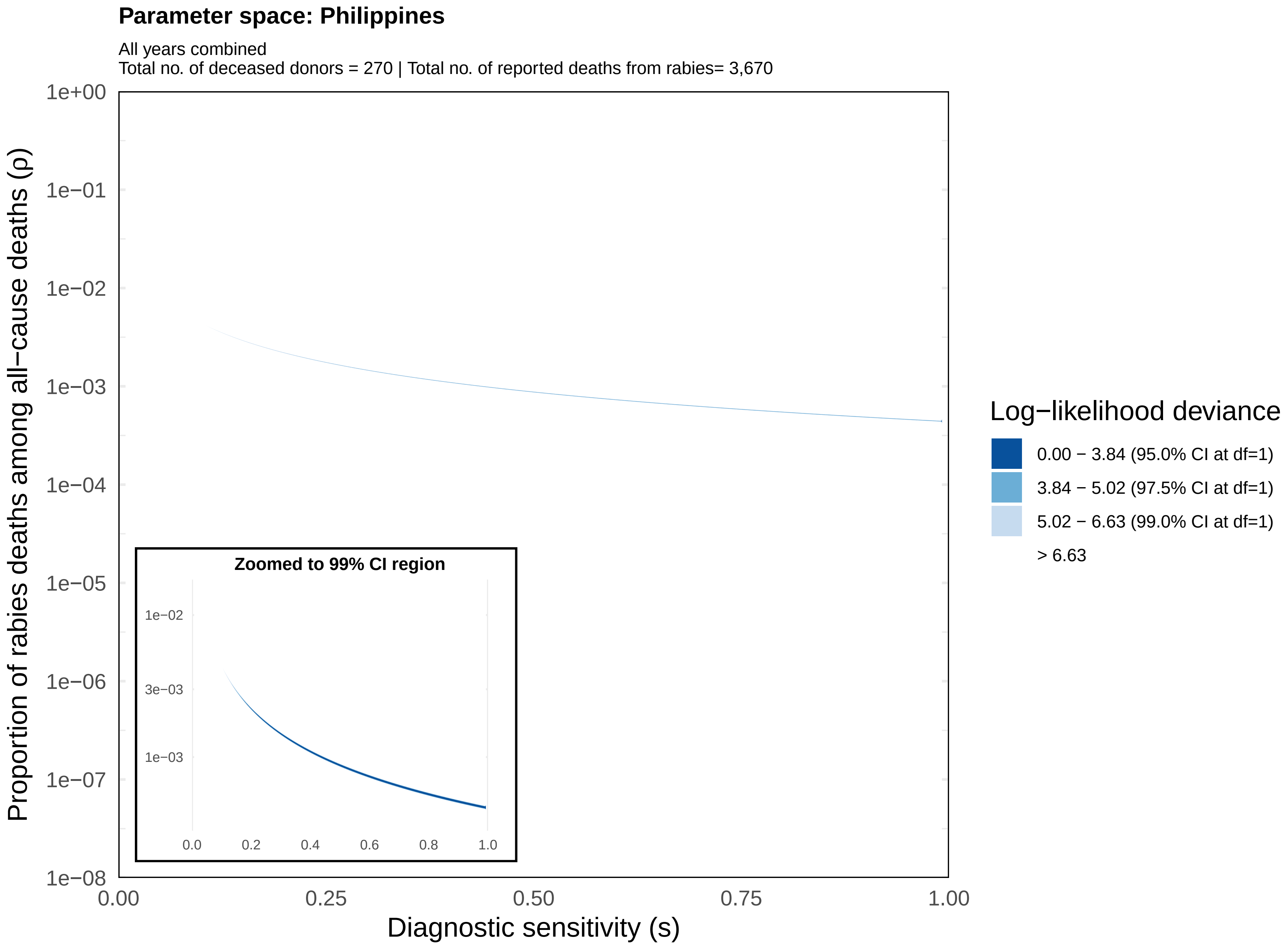

Parameter space: Philippines
All years combined
Total no. of deceased donors = 270 | Total no. of reported deaths from rabies= 3,670
1e+00
1e−01
1e−02
1e−03
1e−04
1e−05
1e−06
1e−07
1e−08
Proportion of rabies deaths among all−cause deaths (ρ)
0.00
0.25
0.50
0.75
1.00
Diagnostic sensitivity (s)
Zoomed to 99% CI region
1e−02
3e−03
1e−03
0.0
0.2
0.4
0.6
0.8
1.0
Log−likelihood deviance
0.00 − 3.84 (95.0% CI at df=1)
3.84 − 5.02 (97.5% CI at df=1)
5.02 − 6.63 (99.0% CI at df=1)
> 6.63

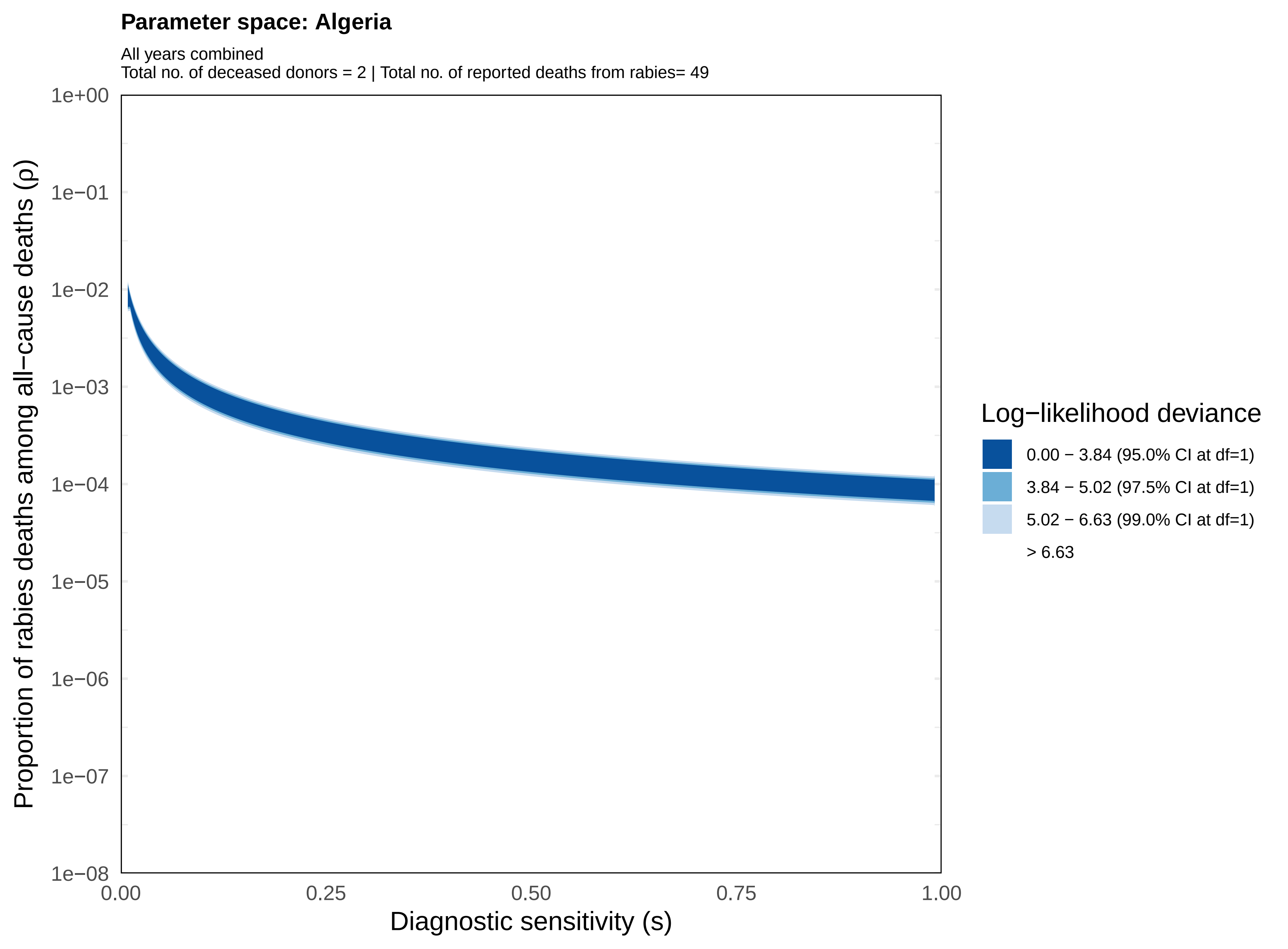

Parameter space: Algeria
All years combined
Total no. of deceased donors = 2 | Total no. of reported deaths from rabies= 49
1e+00
1e−01
1e−02
1e−03
1e−04
1e−05
1e−06
1e−07
1e−08
Proportion of rabies deaths among all−cause deaths (ρ)
0.00
0.25
0.50
0.75
1.00
Diagnostic sensitivity (s)
Log−likelihood deviance
0.00 − 3.84 (95.0% CI at df=1)
3.84 − 5.02 (97.5% CI at df=1)
5.02 − 6.63 (99.0% CI at df=1)
> 6.63

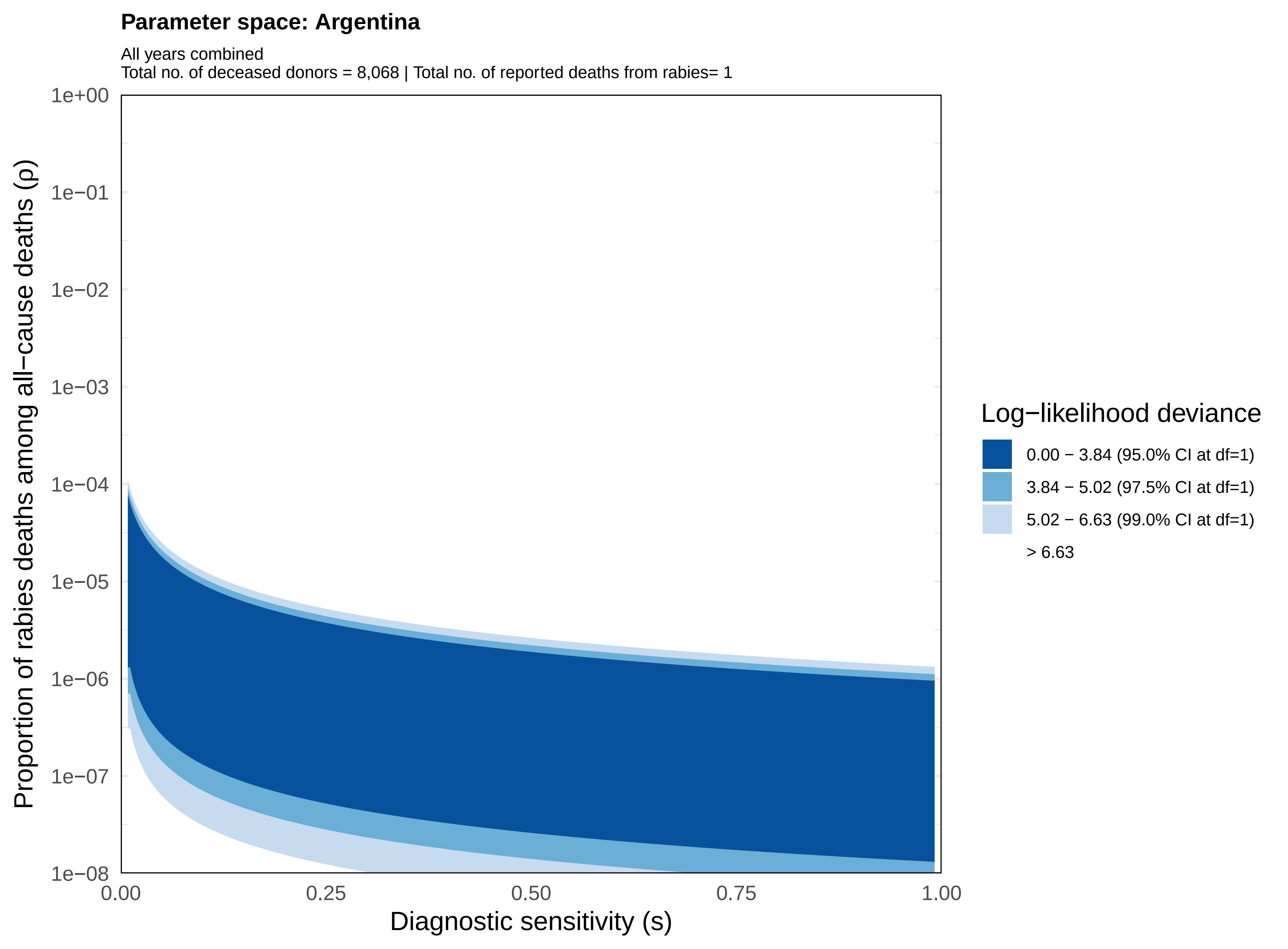

Parameter space: Argentina
All years combined
Total no. of deceased donors = 8,068 | Total no. of reported deaths from rabies= 1
Proportion of rabies deaths among all−cause deaths (ρ)
1e+00
1e−01
1e−02
1e−03
1e−04
1e−05
1e−06
1e−07
1e−08
0.00
0.25
0.50
0.75
1.00
Diagnostic sensitivity (s)
Log−likelihood deviance
0.00 − 3.84 (95.0% CI at df=1)
3.84 − 5.02 (97.5% CI at df=1)
5.02 − 6.63 (99.0% CI at df=1)
> 6.63

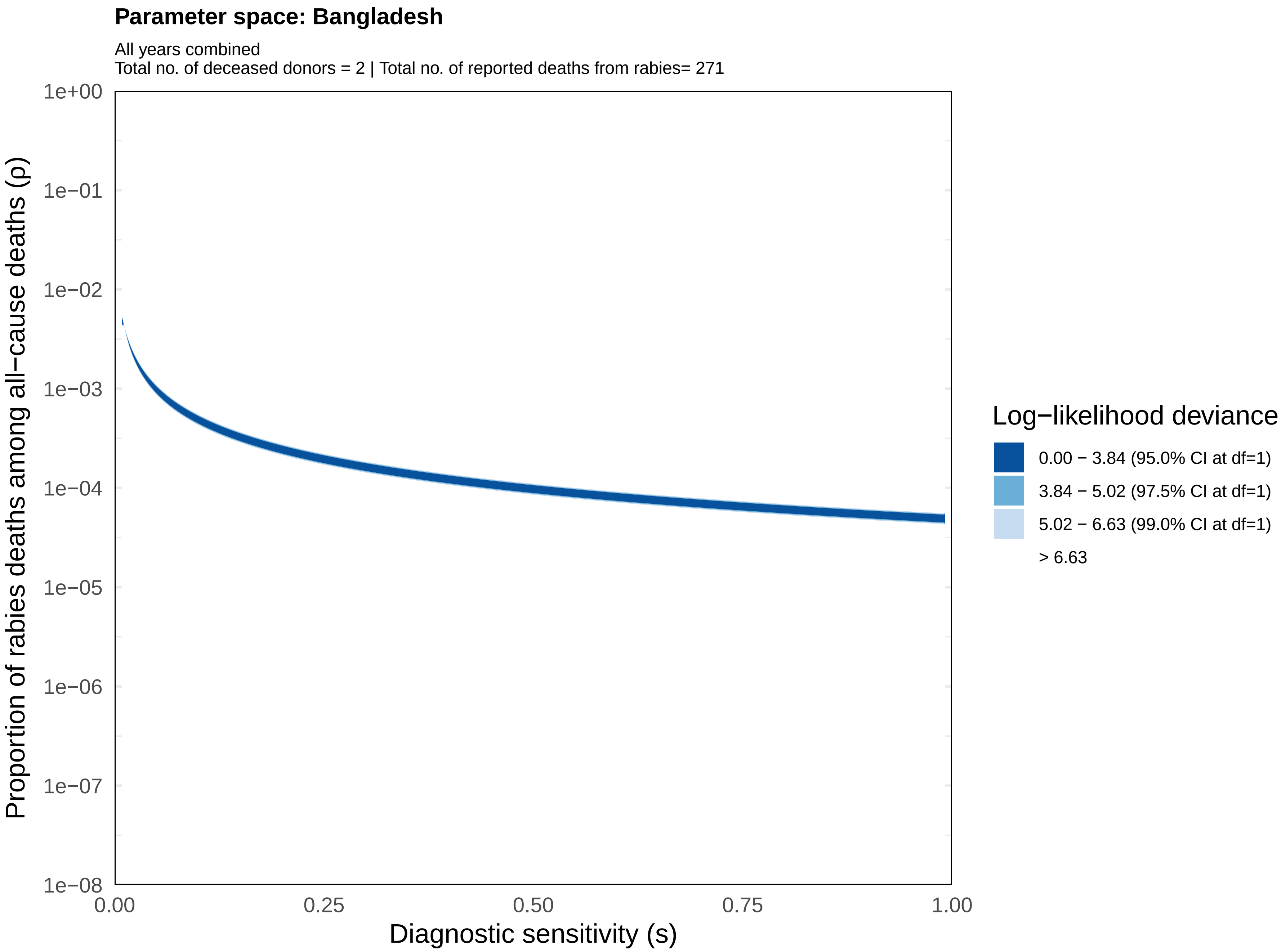
Parameter space: Bangladesh
All years combined
Total no. of deceased donors = 2 | Total no. of reported deaths from rabies= 271
Proportion of rabies deaths among all−cause deaths (ρ)
1e+00
1e−01
1e−02
1e−03
1e−04
1e−05
1e−06
1e−07
1e−08
Diagnostic sensitivity (s)
0.00
0.25
0.50
0.75
1.00
Log−likelihood deviance
0.00 − 3.84 (95.0% CI at df=1)
3.84 − 5.02 (97.5% CI at df=1)
5.02 − 6.63 (99.0% CI at df=1)
> 6.63

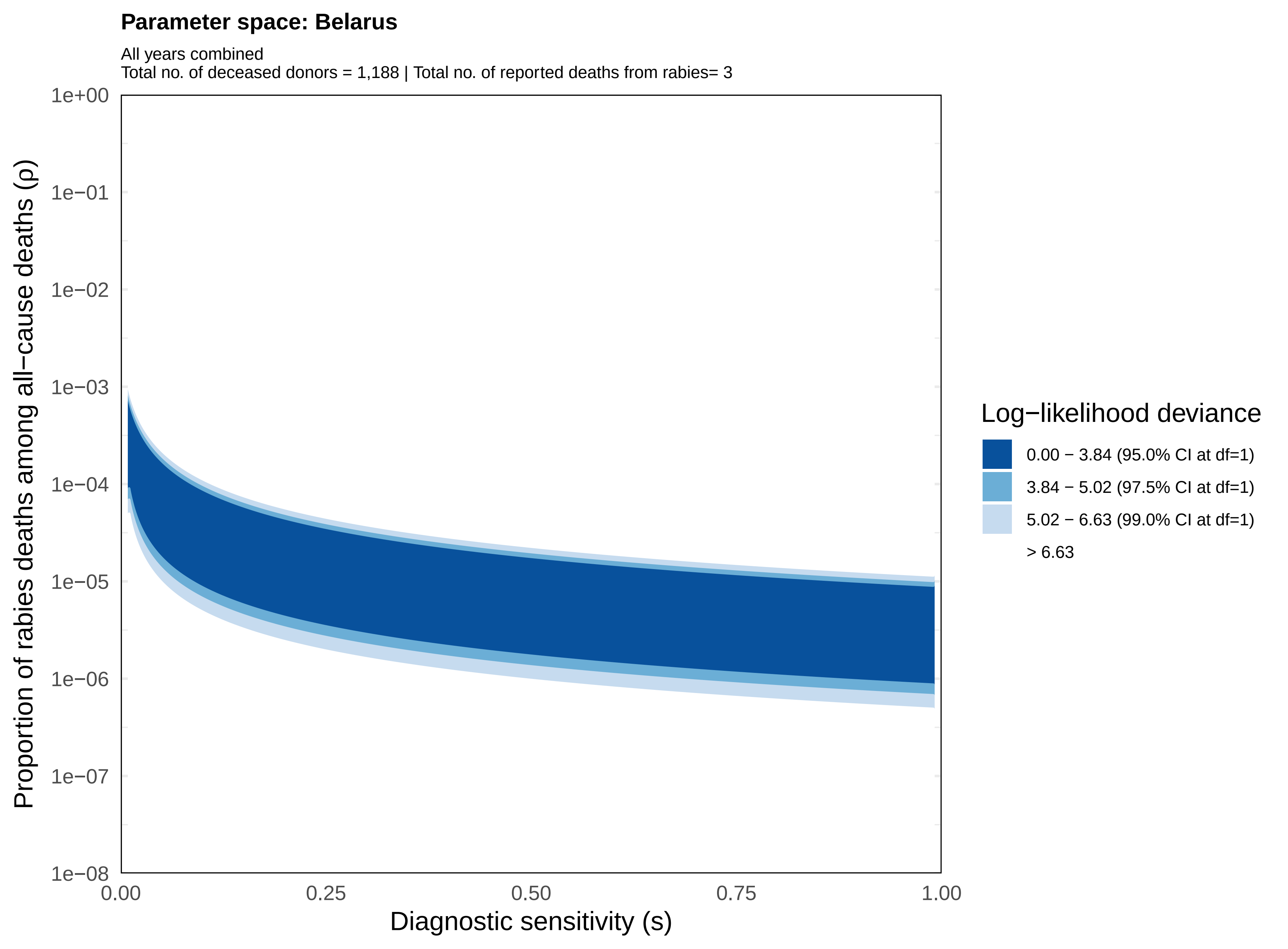

Parameter space: Belarus
All years combined
Total no. of deceased donors = 1,188 | Total no. of reported deaths from rabies= 3
Proportion of rabies deaths among all−cause deaths (ρ)
1e+00
1e−01
1e−02
1e−03
1e−04
1e−05
1e−06
1e−07
1e−08
0.00
0.25
0.50
0.75
1.00
Diagnostic sensitivity (s)
Log−likelihood deviance
0.00 − 3.84 (95.0% CI at df=1)
3.84 − 5.02 (97.5% CI at df=1)
5.02 − 6.63 (99.0% CI at df=1)
> 6.63

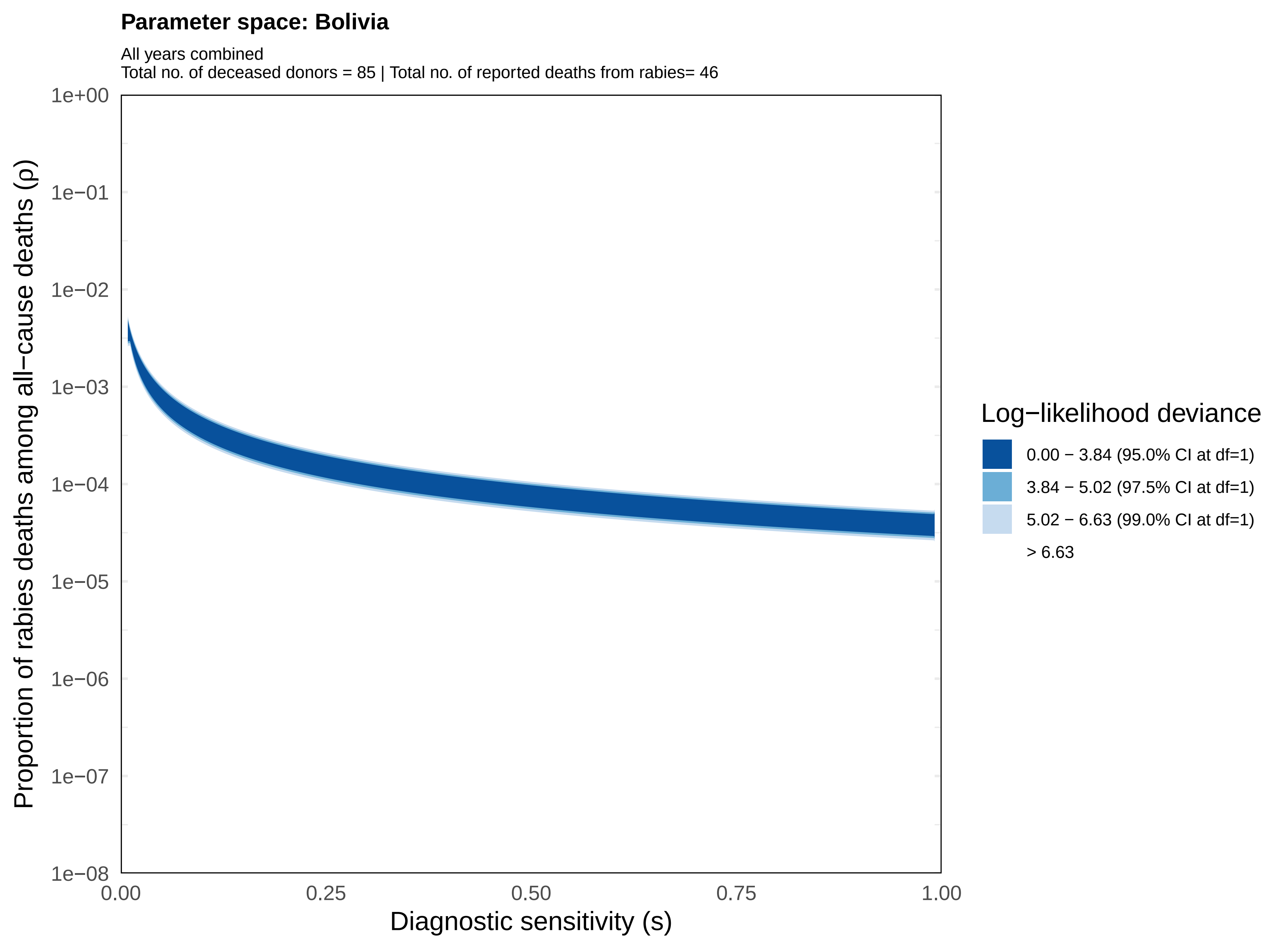

Parameter space: Bolivia
All years combined
Total no. of deceased donors = 85 | Total no. of reported deaths from rabies= 46
Proportion of rabies deaths among all−cause deaths (ρ)
1e+00
1e−01
1e−02
1e−03
1e−04
1e−05
1e−06
1e−07
1e−08
0.00
0.25
0.50
0.75
1.00
Diagnostic sensitivity (s)
Log−likelihood deviance
0.00 − 3.84 (95.0% CI at df=1)
3.84 − 5.02 (97.5% CI at df=1)
5.02 − 6.63 (99.0% CI at df=1)
> 6.63

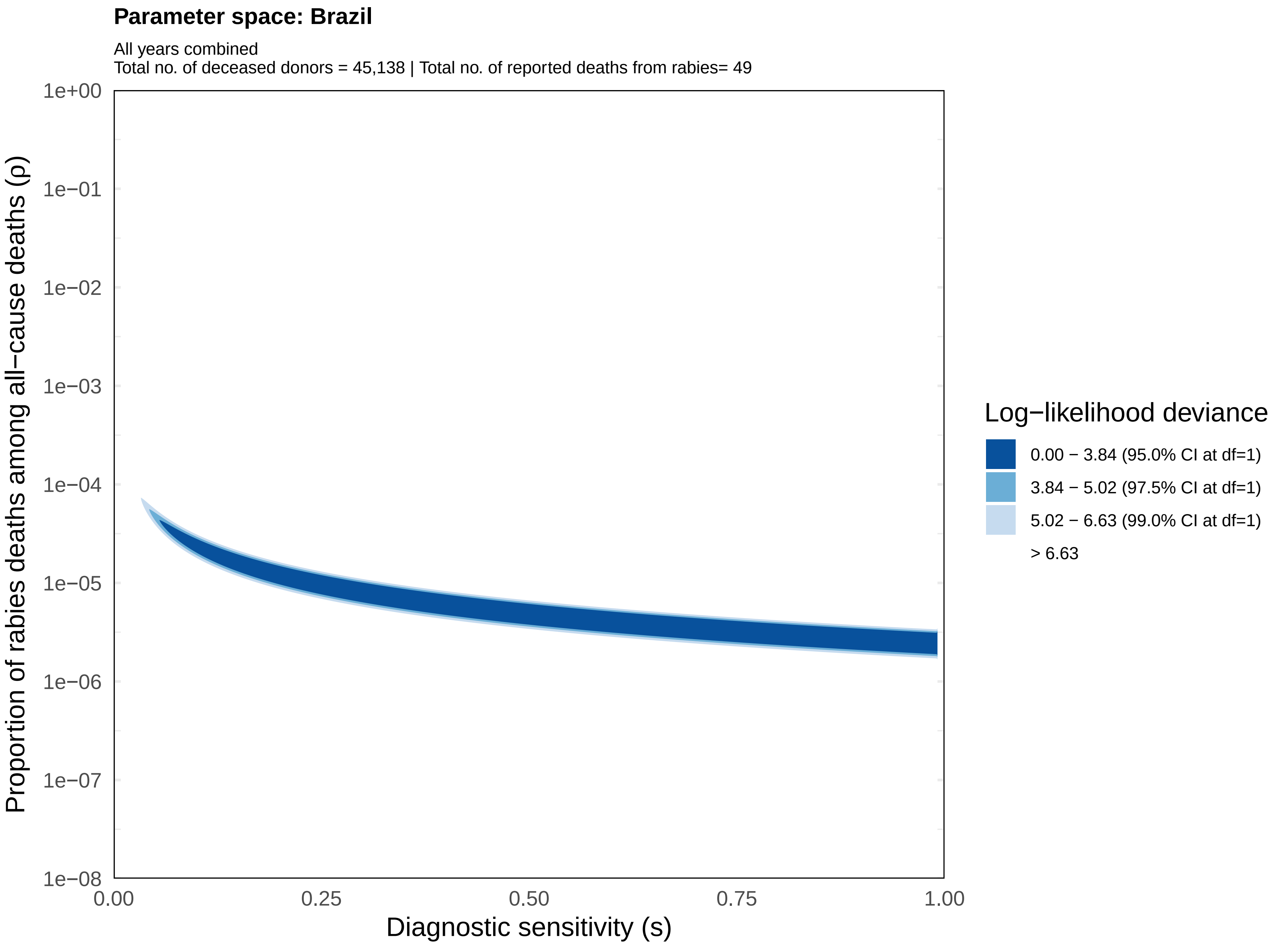
Parameter space: Brazil
All years combined
Total no. of deceased donors = 45,138 | Total no. of reported deaths from rabies= 49
1e+00
1e−01
1e−02
1e−03
1e−04
1e−05
1e−06
1e−07
1e−08
Proportion of rabies deaths among all−cause deaths (ρ)
0.00
0.25
0.50
0.75
1.00
Diagnostic sensitivity (s)
Log−likelihood deviance
0.00 − 3.84 (95.0% CI at df=1)
3.84 − 5.02 (97.5% CI at df=1)
5.02 − 6.63 (99.0% CI at df=1)
> 6.63

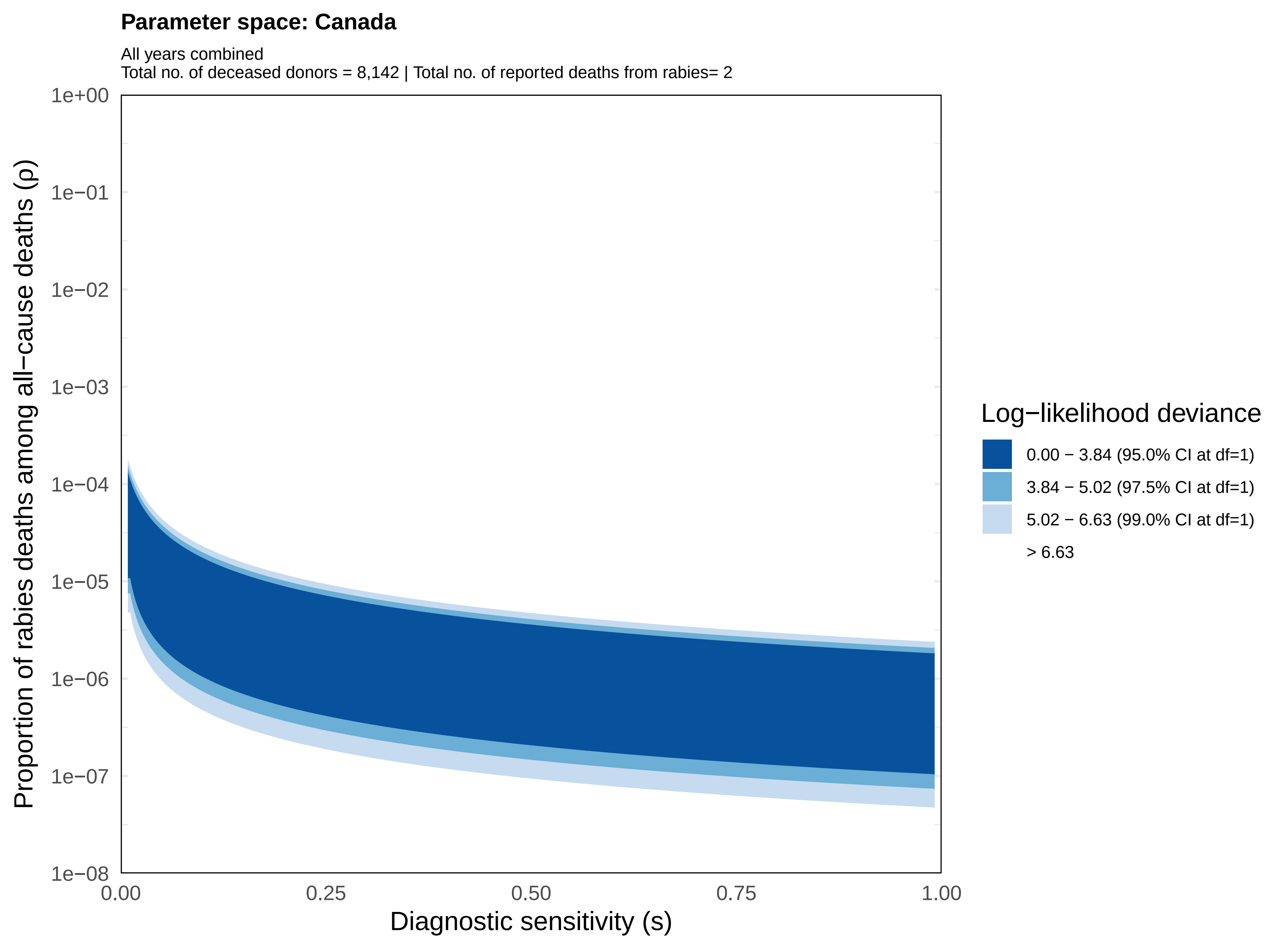

Parameter space: Canada
All years combined
Total no. of deceased donors = 8,142 | Total no. of reported deaths from rabies= 2
Proportion of rabies deaths among all−cause deaths (ρ)
1e+00
1e−01
1e−02
1e−03
1e−04
1e−05
1e−06
1e−07
1e−08
0.00
0.25
0.50
0.75
1.00
Diagnostic sensitivity (s)
Log−likelihood deviance
0.00 − 3.84 (95.0% CI at df=1)
3.84 − 5.02 (97.5% CI at df=1)
5.02 − 6.63 (99.0% CI at df=1)
> 6.63

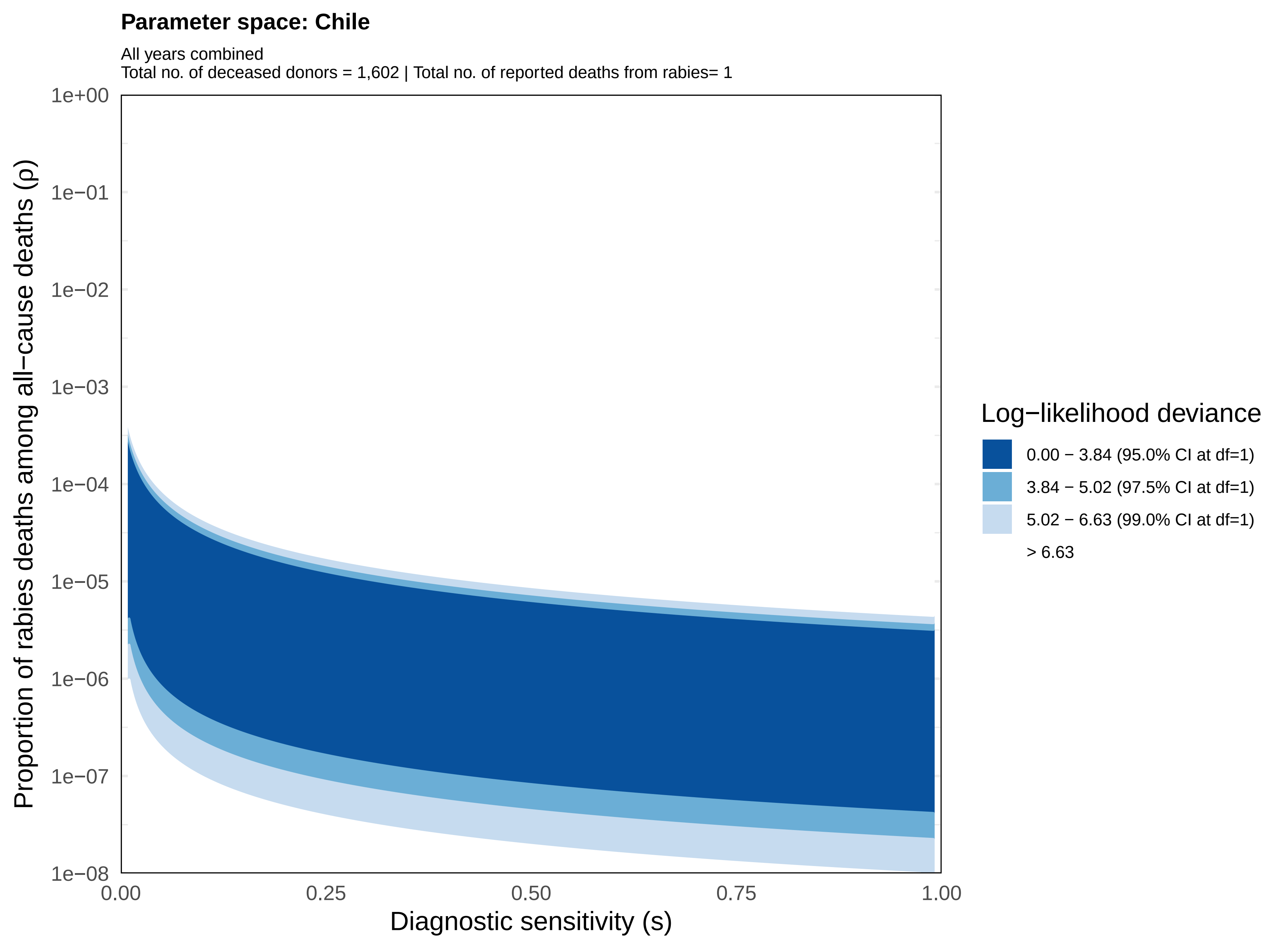
Parameter space: Chile
All years combined
Total no. of deceased donors = 1,602 | Total no. of reported deaths from rabies= 1
Proportion of rabies deaths among all−cause deaths (ρ)
1e+00
1e−01
1e−02
1e−03
1e−04
1e−05
1e−06
1e−07
1e−08
0.00
0.25
0.50
0.75
1.00
Diagnostic sensitivity (s)
Log−likelihood deviance
0.00 − 3.84 (95.0% CI at df=1)
3.84 − 5.02 (97.5% CI at df=1)
5.02 − 6.63 (99.0% CI at df=1)
> 6.63

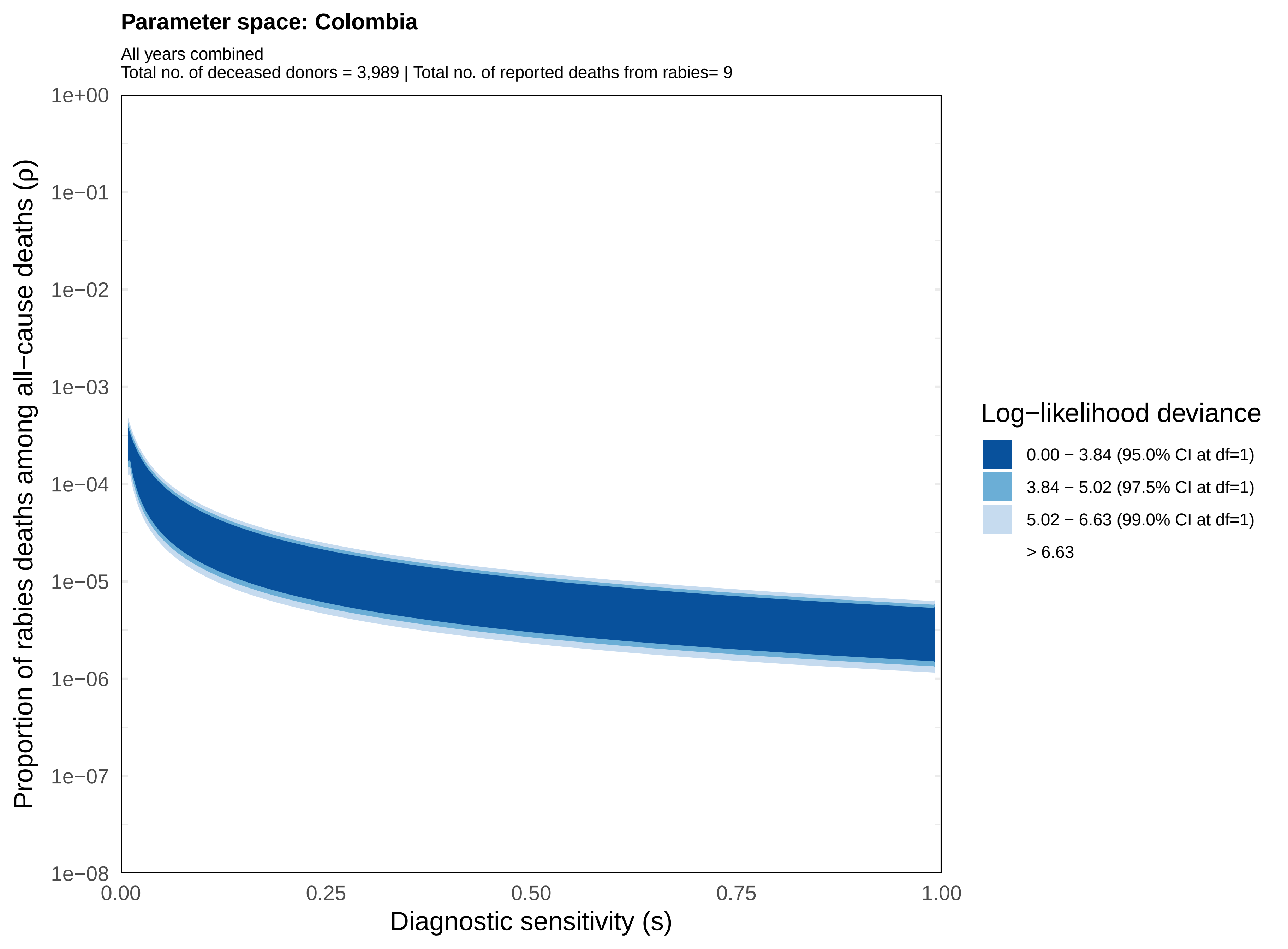
Parameter space: Colombia
All years combined
Total no. of deceased donors = 3,989 | Total no. of reported deaths from rabies= 9
1e+00
1e−01
1e−02
1e−03
1e−04
1e−05
1e−06
1e−07
1e−08
Proportion of rabies deaths among all−cause deaths (ρ)
0.00
0.25
0.50
0.75
1.00
Diagnostic sensitivity (s)
Log−likelihood deviance
0.00 − 3.84 (95.0% CI at df=1)
3.84 − 5.02 (97.5% CI at df=1)
5.02 − 6.63 (99.0% CI at df=1)
> 6.63

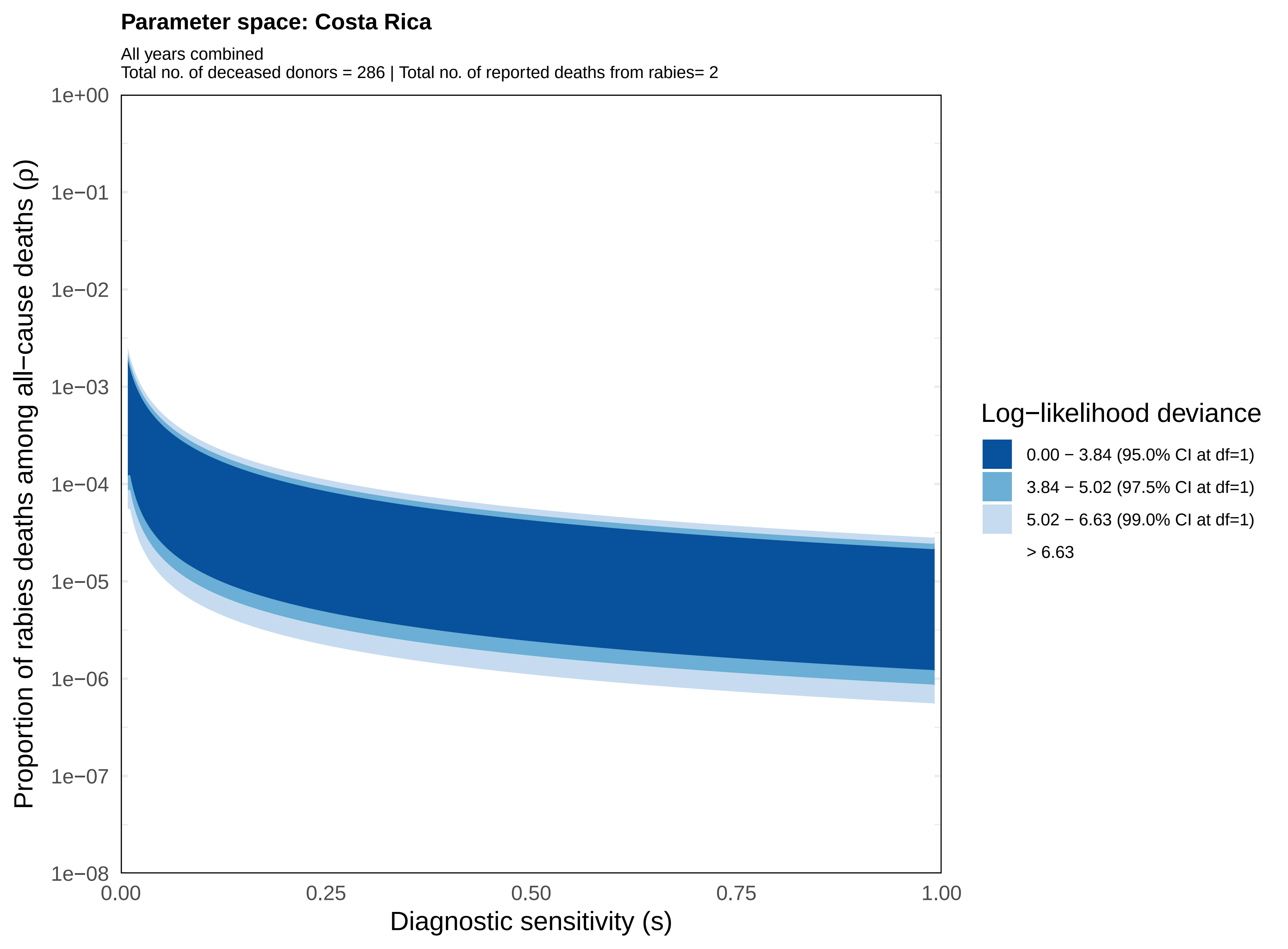

Parameter space: Costa Rica
All years combined
Total no. of deceased donors = 286 | Total no. of reported deaths from rabies= 2
1e+00
1e−01
1e−02
1e−03
1e−04
1e−05
1e−06
1e−07
1e−08
Proportion of rabies deaths among all−cause deaths (ρ)
0.00
0.25
0.50
0.75
1.00
Diagnostic sensitivity (s)
Log−likelihood deviance
0.00 − 3.84 (95.0% CI at df=1)
3.84 − 5.02 (97.5% CI at df=1)
5.02 − 6.63 (99.0% CI at df=1)
> 6.63

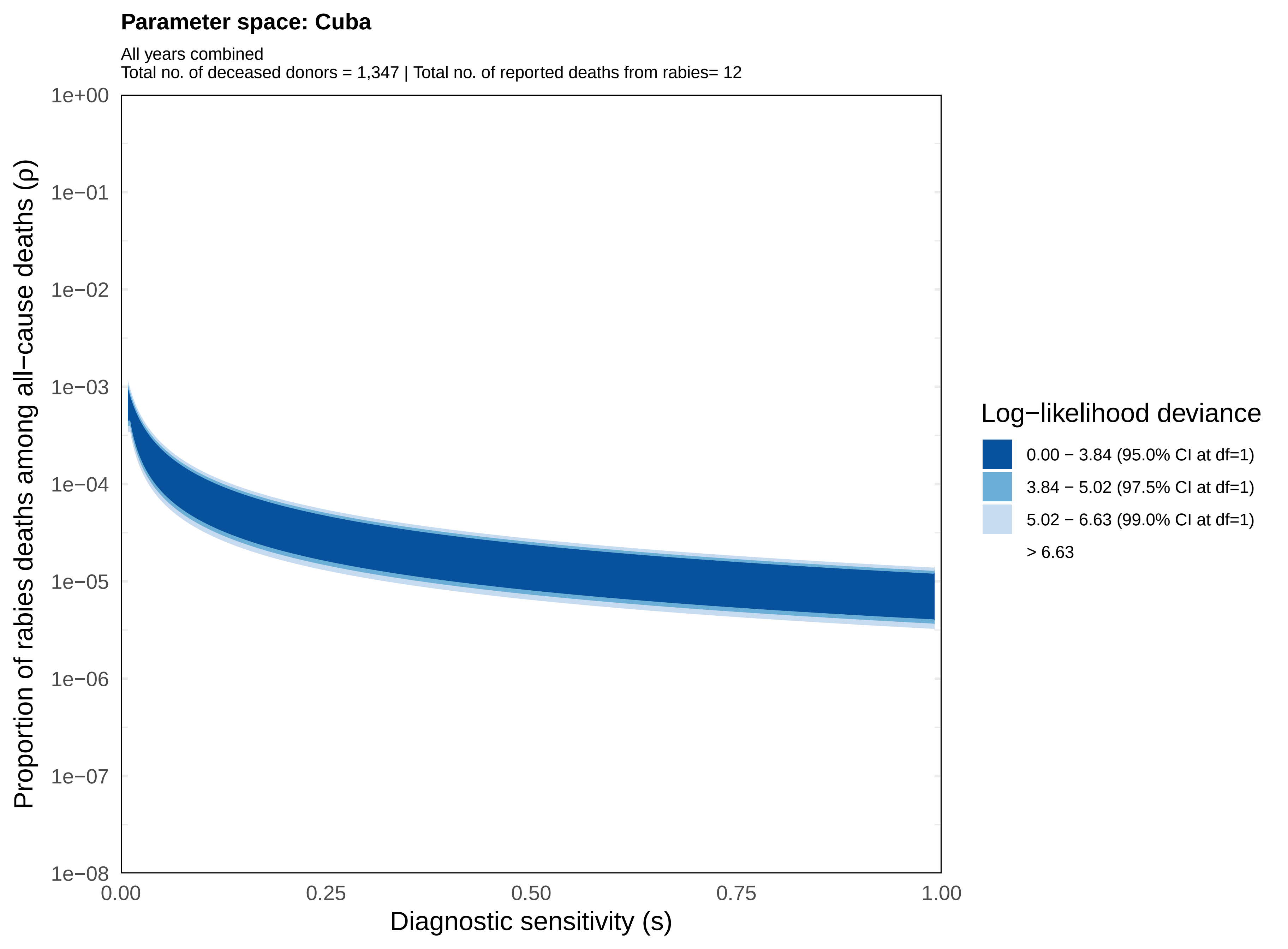
Parameter space: Cuba
All years combined
Total no. of deceased donors = 1,347 | Total no. of reported deaths from rabies= 12
1e+00
1e−01
1e−02
1e−03
1e−04
1e−05
1e−06
1e−07
1e−08
Proportion of rabies deaths among all−cause deaths (ρ)
0.00
0.25
0.50
0.75
1.00
Diagnostic sensitivity (s)
Log−likelihood deviance
0.00 − 3.84 (95.0% CI at df=1)
3.84 − 5.02 (97.5% CI at df=1)
5.02 − 6.63 (99.0% CI at df=1)
> 6.63

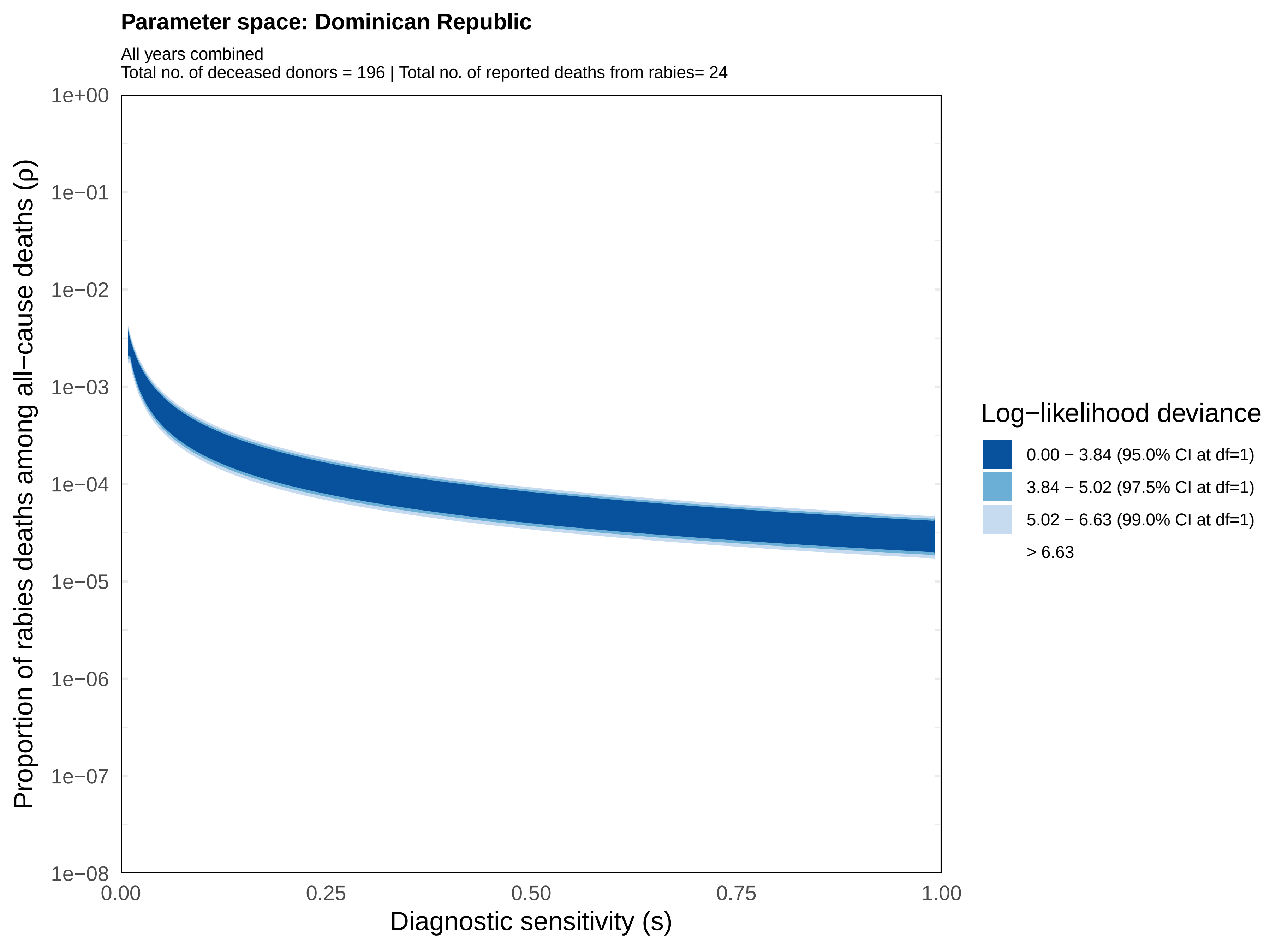
Parameter space: Dominican Republic
All years combined
Total no. of deceased donors = 196 | Total no. of reported deaths from rabies= 24
1e+00
1e−01
1e−02
1e−03
1e−04
1e−05
1e−06
1e−07
1e−08
Proportion of rabies deaths among all−cause deaths (ρ)
0.00
0.25
0.50
0.75
1.00
Diagnostic sensitivity (s)
Log−likelihood deviance
0.00 − 3.84 (95.0% CI at df=1)
3.84 − 5.02 (97.5% CI at df=1)
5.02 − 6.63 (99.0% CI at df=1)
> 6.63

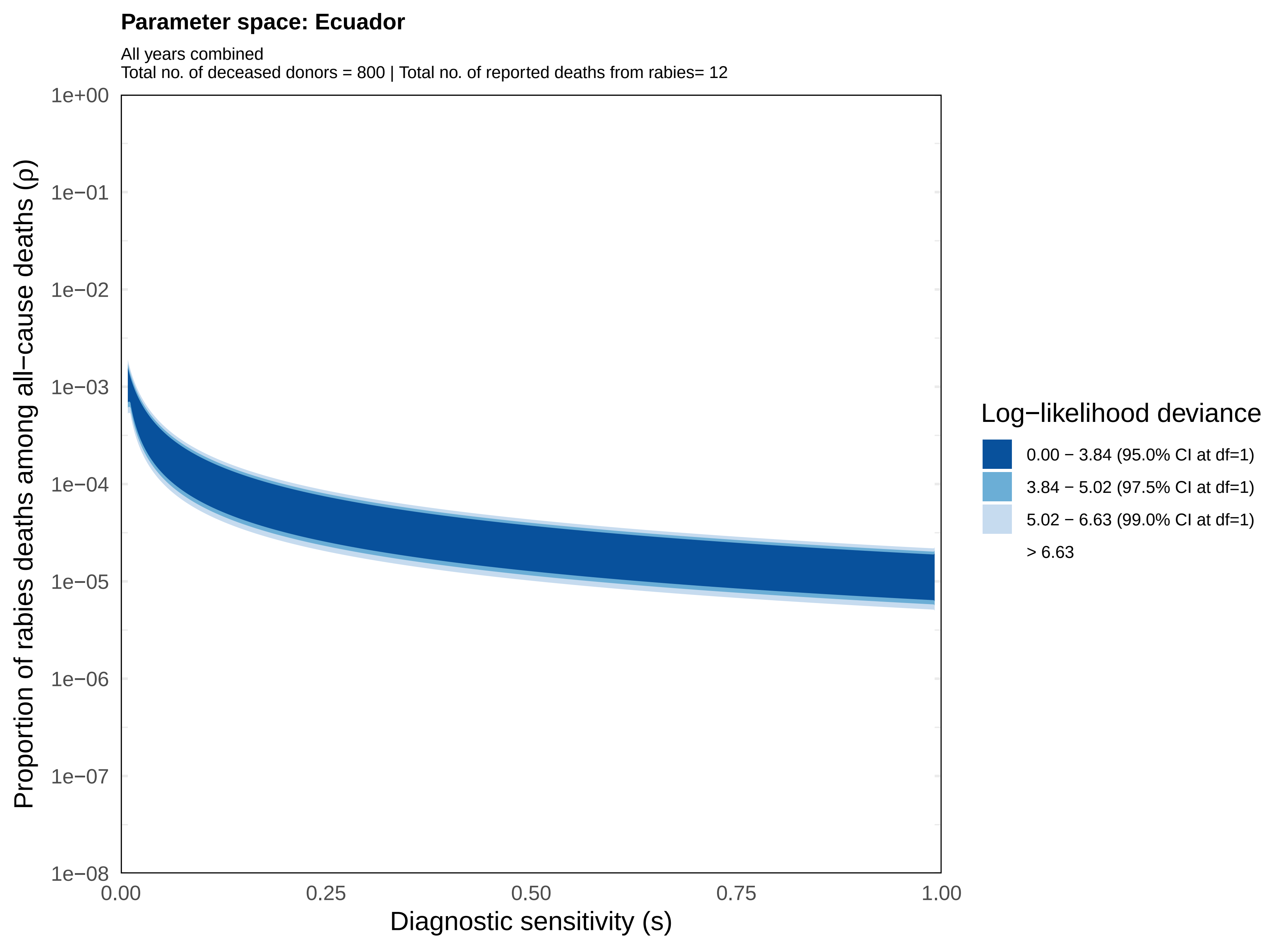

Parameter space: Ecuador
All years combined
Total no. of deceased donors = 800 | Total no. of reported deaths from rabies= 12
Proportion of rabies deaths among all−cause deaths (ρ)
1e+00
1e−01
1e−02
1e−03
1e−04
1e−05
1e−06
1e−07
1e−08
Diagnostic sensitivity (s)
0.00
0.25
0.50
0.75
1.00
Log−likelihood deviance
0.00 − 3.84 (95.0% CI at df=1)
3.84 − 5.02 (97.5% CI at df=1)
5.02 − 6.63 (99.0% CI at df=1)
> 6.63

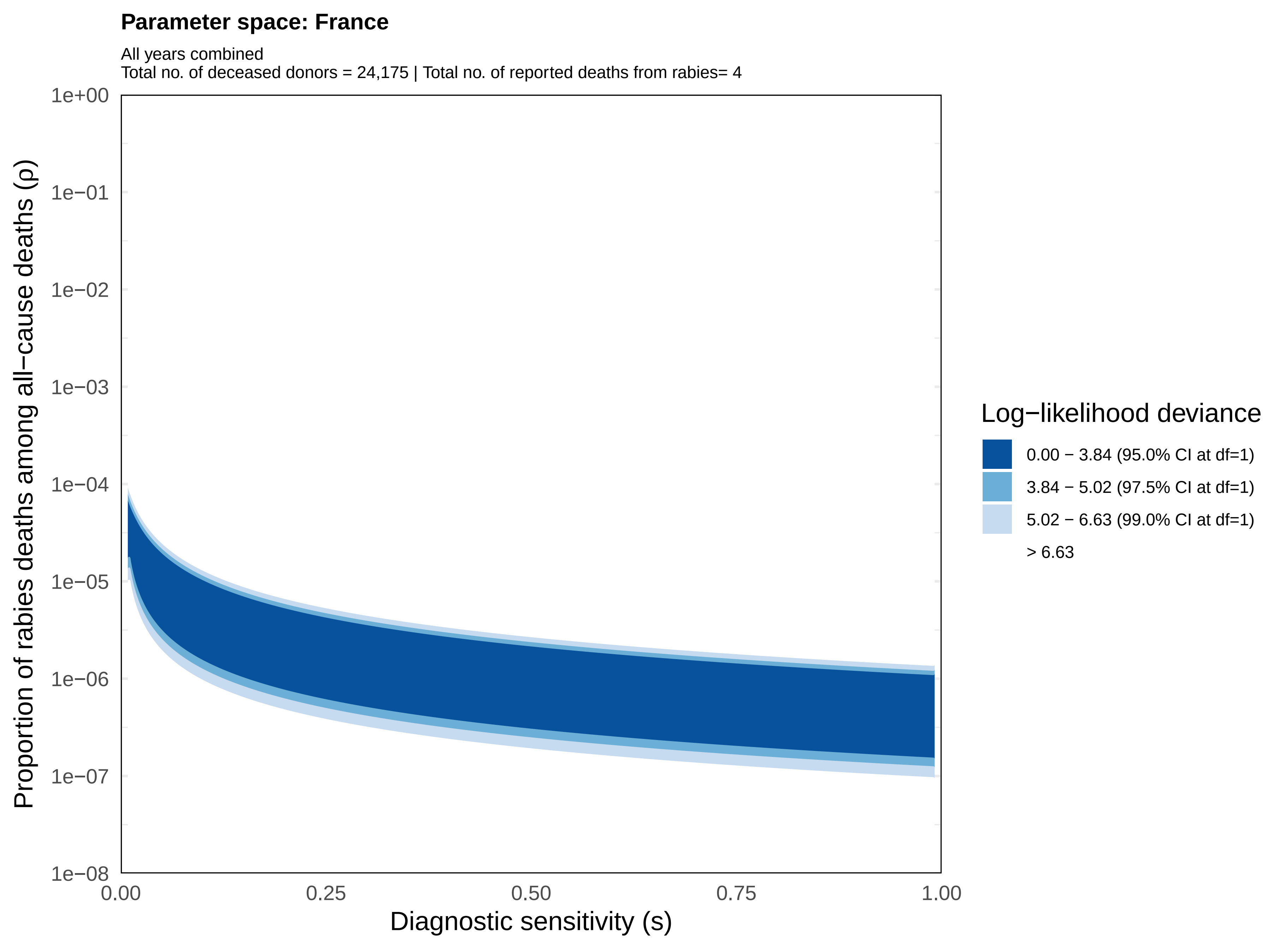

Parameter space: France
All years combined
Total no. of deceased donors = 24,175 | Total no. of reported deaths from rabies= 4
Proportion of rabies deaths among all−cause deaths (ρ)
1e+00
1e−01
1e−02
1e−03
1e−04
1e−05
1e−06
1e−07
1e−08
0.00
0.25
0.50
0.75
1.00
Diagnostic sensitivity (s)
Log−likelihood deviance
0.00 − 3.84 (95.0% CI at df=1)
3.84 − 5.02 (97.5% CI at df=1)
5.02 − 6.63 (99.0% CI at df=1)
> 6.63

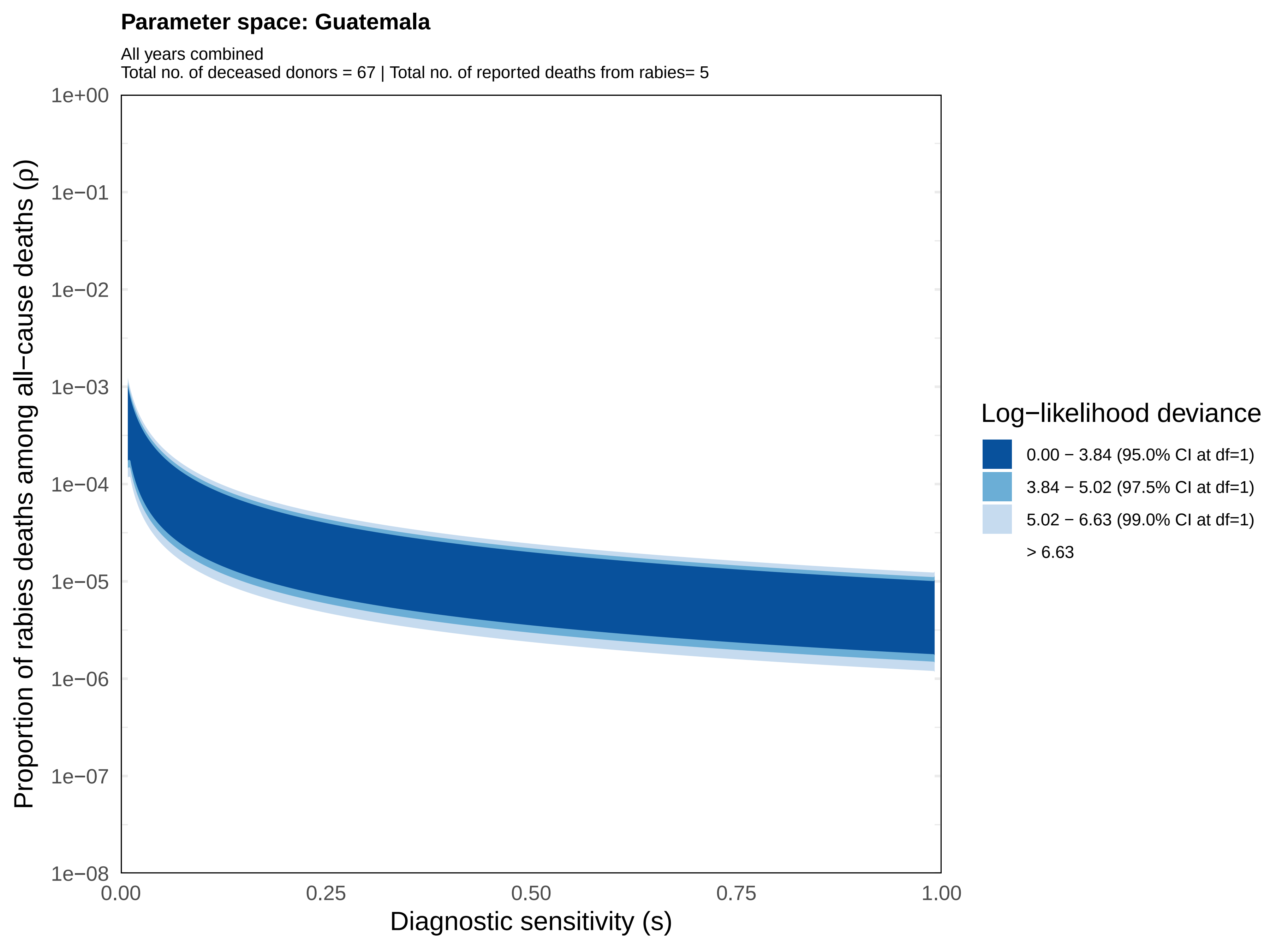

Parameter space: Guatemala
All years combined
Total no. of deceased donors = 67 | Total no. of reported deaths from rabies= 5
Proportion of rabies deaths among all−cause deaths (ρ)
1e+00
1e−01
1e−02
1e−03
1e−04
1e−05
1e−06
1e−07
1e−08
0.00
0.25
0.50
0.75
1.00
Diagnostic sensitivity (s)
Log−likelihood deviance
0.00 − 3.84 (95.0% CI at df=1)
3.84 − 5.02 (97.5% CI at df=1)
5.02 − 6.63 (99.0% CI at df=1)
> 6.63

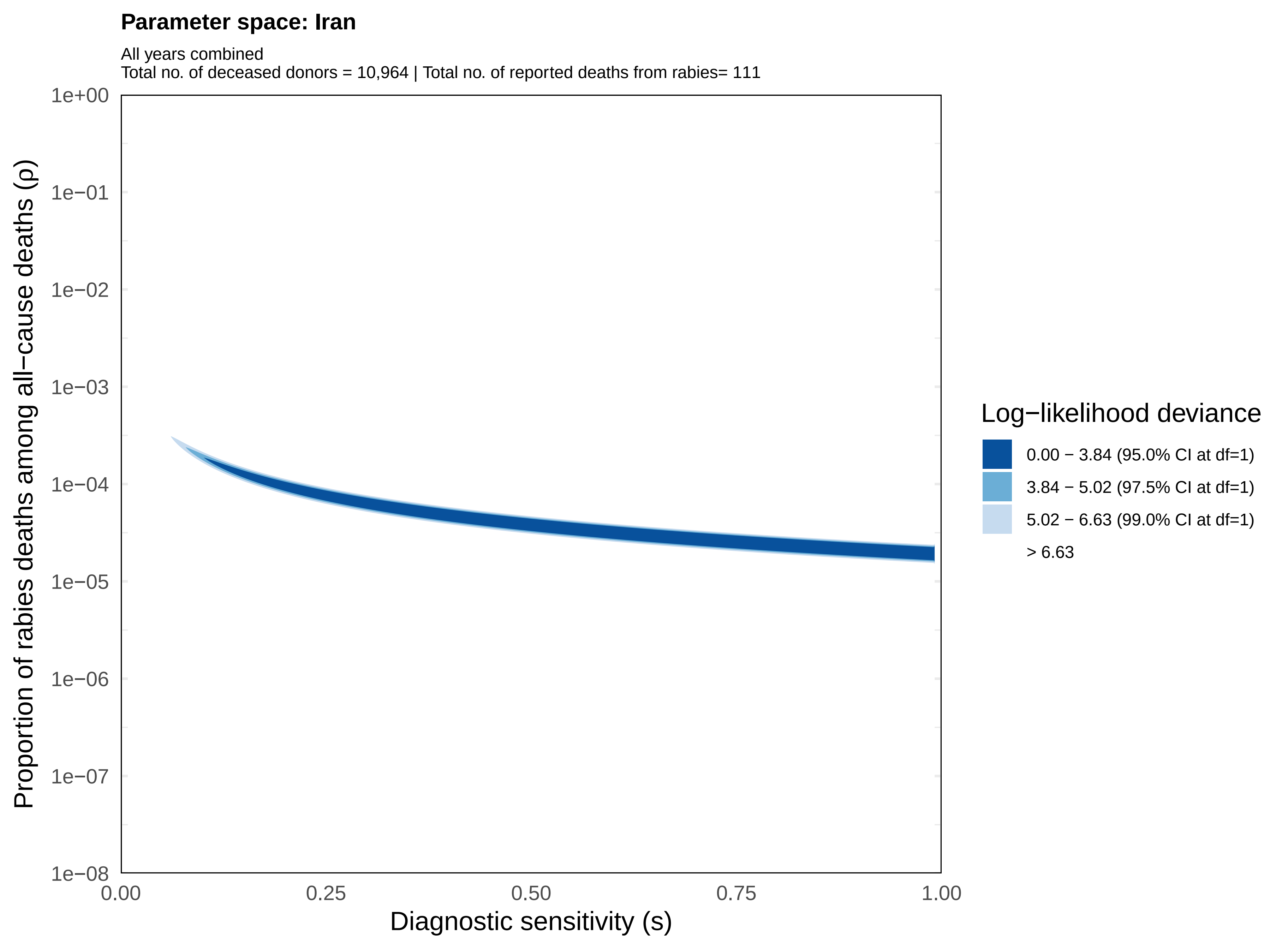

Parameter space: Iran
All years combined
Total no. of deceased donors = 10,964 | Total no. of reported deaths from rabies= 111
Proportion of rabies deaths among all−cause deaths (ρ)
1e+00
1e−01
1e−02
1e−03
1e−04
1e−05
1e−06
1e−07
1e−08
0.00
0.25
0.50
0.75
1.00
Diagnostic sensitivity (s)
Log−likelihood deviance
0.00 − 3.84 (95.0% CI at df=1)
3.84 − 5.02 (97.5% CI at df=1)
5.02 − 6.63 (99.0% CI at df=1)
> 6.63

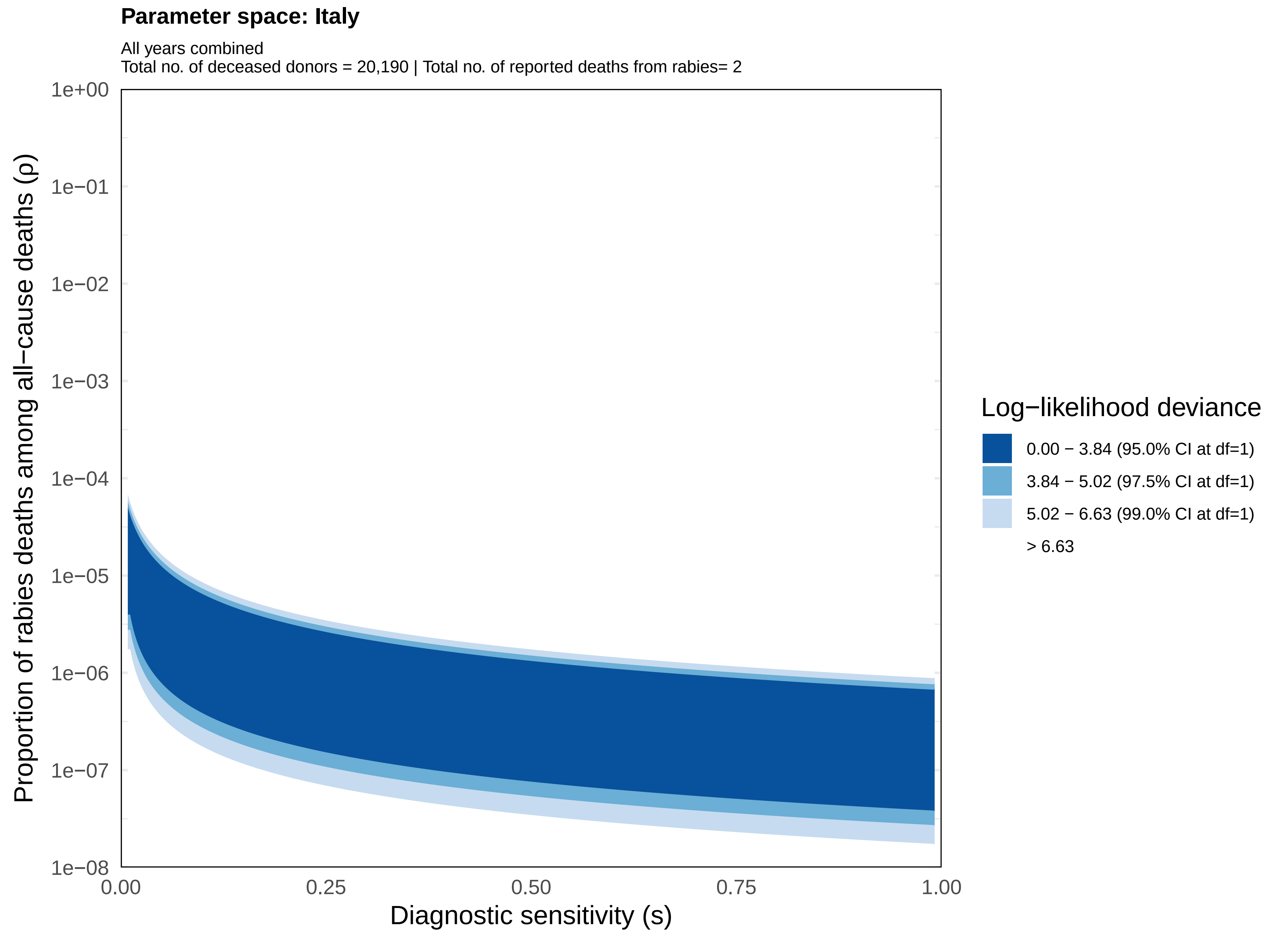
Parameter space: Italy
All years combined
Total no. of deceased donors = 20,190 | Total no. of reported deaths from rabies= 2
1e+00
1e−01
1e−02
1e−03
1e−04
1e−05
1e−06
1e−07
1e−08
Proportion of rabies deaths among all−cause deaths (ρ)
0.00
0.25
0.50
0.75
1.00
Diagnostic sensitivity (s)
Log−likelihood deviance
0.00 − 3.84 (95.0% CI at df=1)
3.84 − 5.02 (97.5% CI at df=1)
5.02 − 6.63 (99.0% CI at df=1)
> 6.63

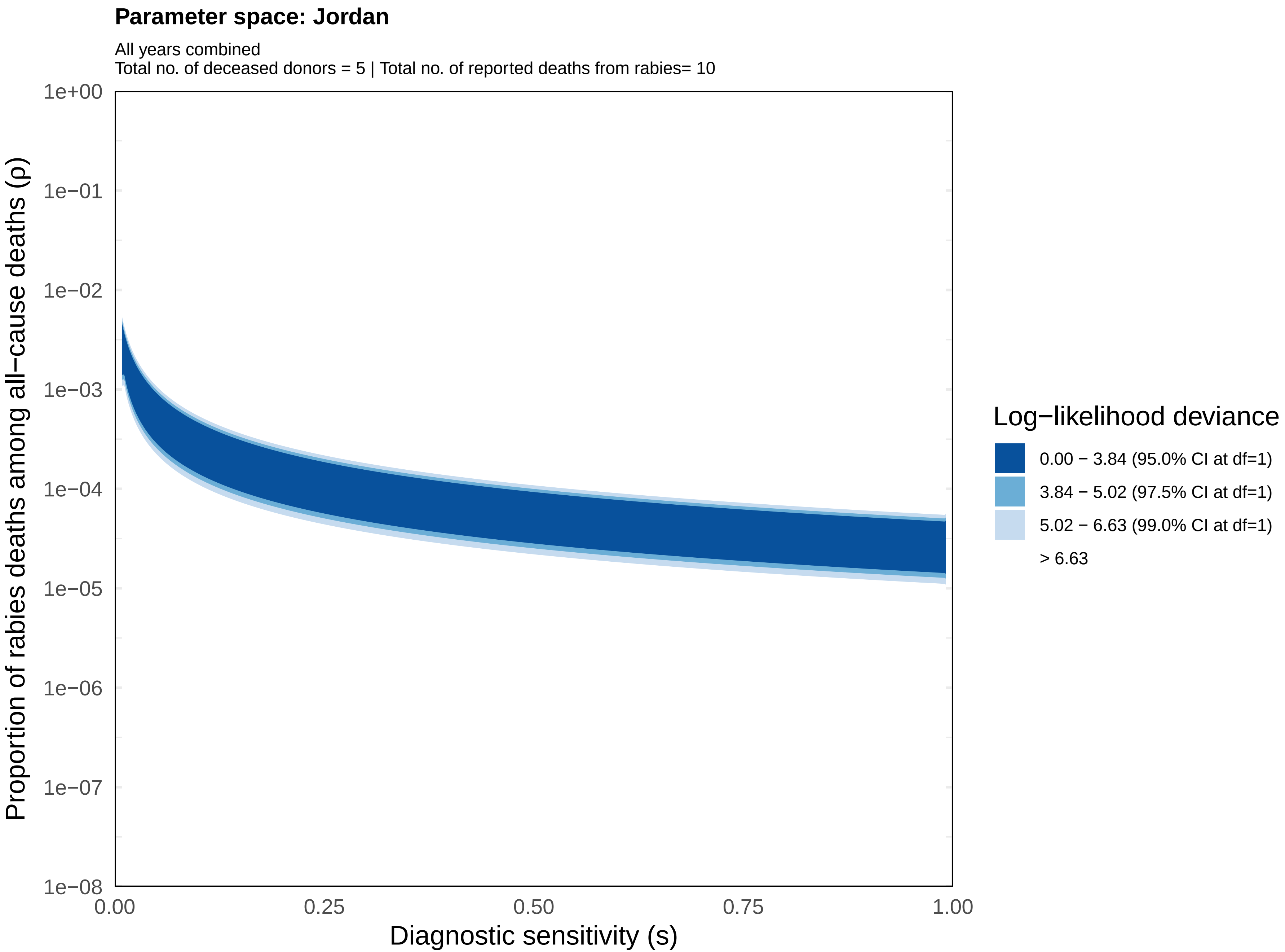
Parameter space: Jordan
All years combined
Total no. of deceased donors = 5 | Total no. of reported deaths from rabies= 10
Proportion of rabies deaths among all−cause deaths (ρ)
1e+00
1e−01
1e−02
1e−03
1e−04
1e−05
1e−06
1e−07
1e−08
0.00
0.25
0.50
0.75
1.00
Diagnostic sensitivity (s)
Log−likelihood deviance
0.00 − 3.84 (95.0% CI at df=1)
3.84 − 5.02 (97.5% CI at df=1)
5.02 − 6.63 (99.0% CI at df=1)
> 6.63

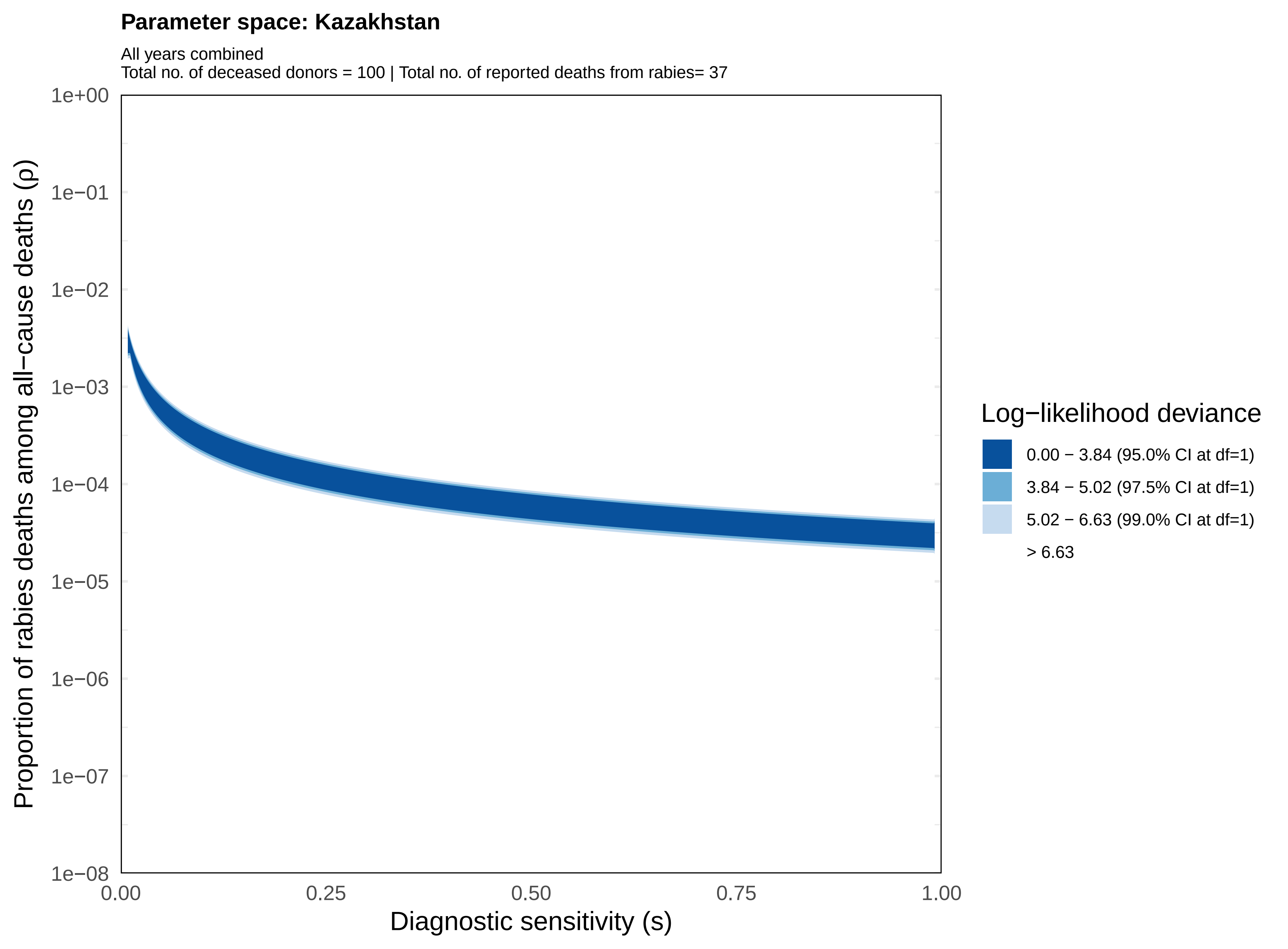

Parameter space: Kazakhstan
All years combined
Total no. of deceased donors = 100 | Total no. of reported deaths from rabies= 37
1e+00
1e−01
1e−02
1e−03
1e−04
1e−05
1e−06
1e−07
1e−08
Proportion of rabies deaths among all−cause deaths (ρ)
0.00
0.25
0.50
0.75
1.00
Diagnostic sensitivity (s)
Log−likelihood deviance
0.00 − 3.84 (95.0% CI at df=1)
3.84 − 5.02 (97.5% CI at df=1)
5.02 − 6.63 (99.0% CI at df=1)
> 6.63

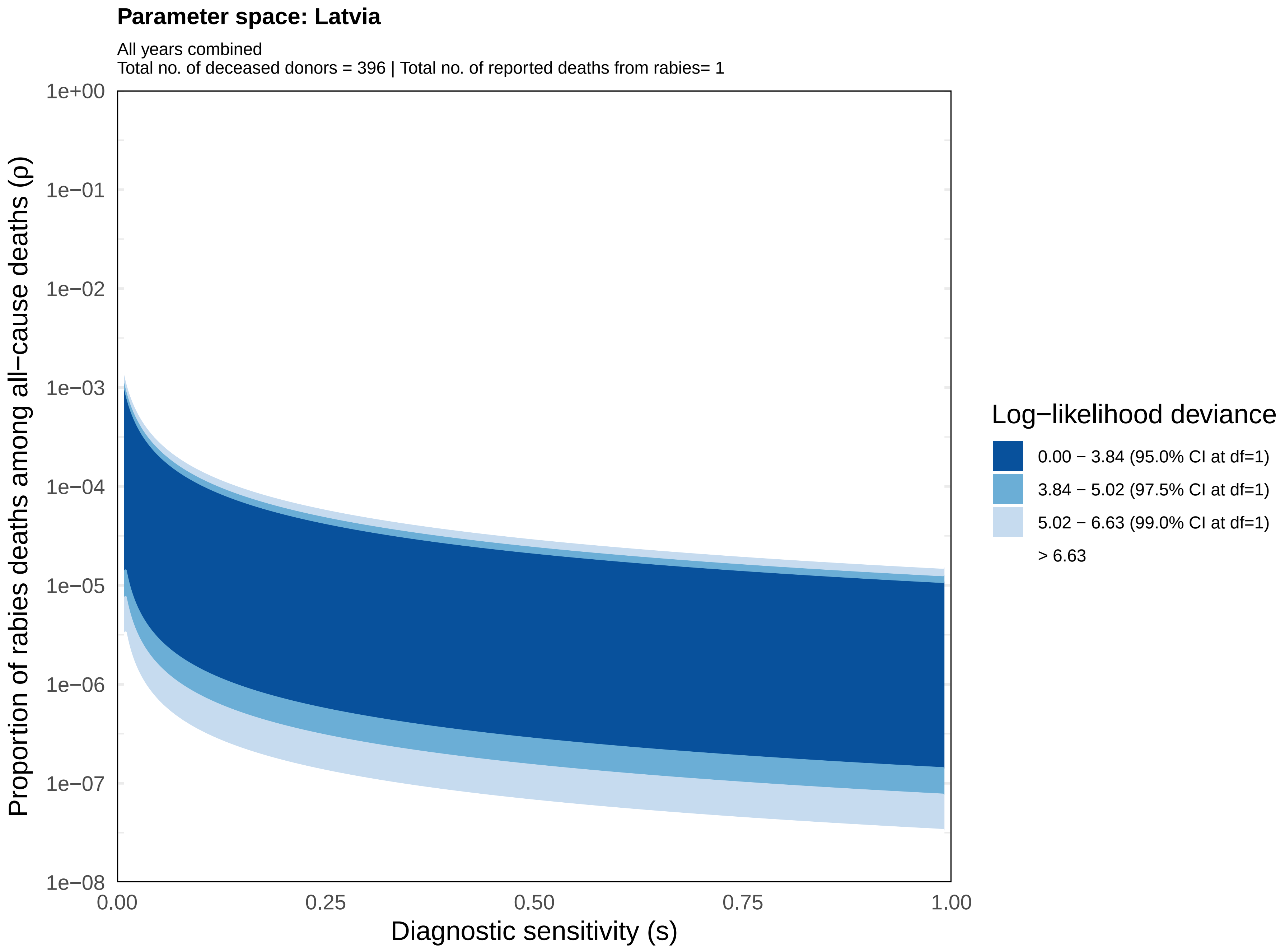

Parameter space: Latvia
All years combined
Total no. of deceased donors = 396 | Total no. of reported deaths from rabies= 1
1e+00
1e−01
1e−02
1e−03
1e−04
1e−05
1e−06
1e−07
1e−08
Proportion of rabies deaths among all−cause deaths (ρ)
0.00
0.25
0.50
0.75
1.00
Diagnostic sensitivity (s)
Log−likelihood deviance
0.00 − 3.84 (95.0% CI at df=1)
3.84 − 5.02 (97.5% CI at df=1)
5.02 − 6.63 (99.0% CI at df=1)
> 6.63

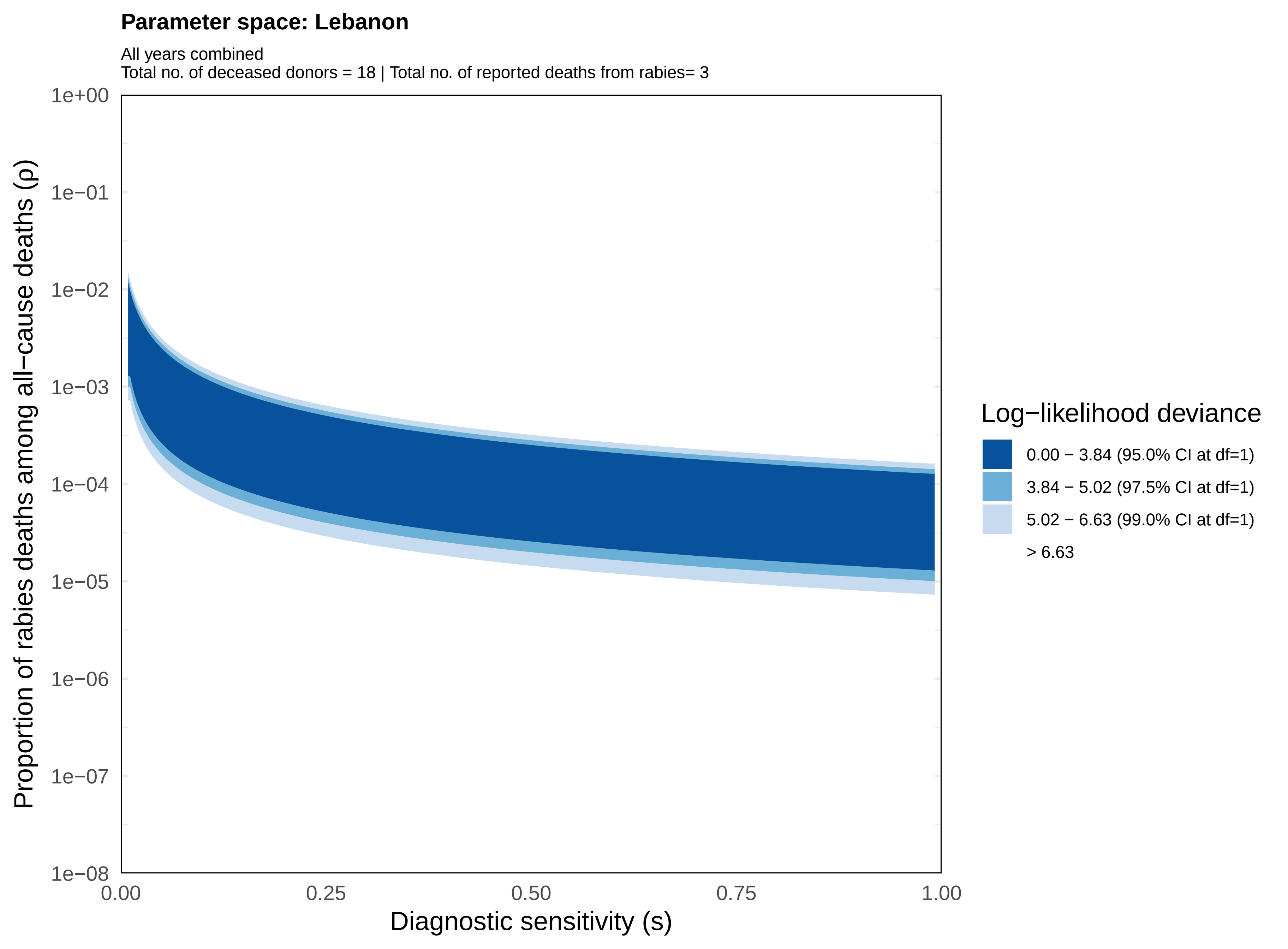
Parameter space: Lebanon
All years combined
Total no. of deceased donors = 18 | Total no. of reported deaths from rabies= 3
1e+00
1e−01
1e−02
1e−03
1e−04
1e−05
1e−06
1e−07
1e−08
Proportion of rabies deaths among all−cause deaths (ρ)
0.00
0.25
0.50
0.75
1.00
Diagnostic sensitivity (s)
Log−likelihood deviance
0.00 − 3.84 (95.0% CI at df=1)
3.84 − 5.02 (97.5% CI at df=1)
5.02 − 6.63 (99.0% CI at df=1)
> 6.63

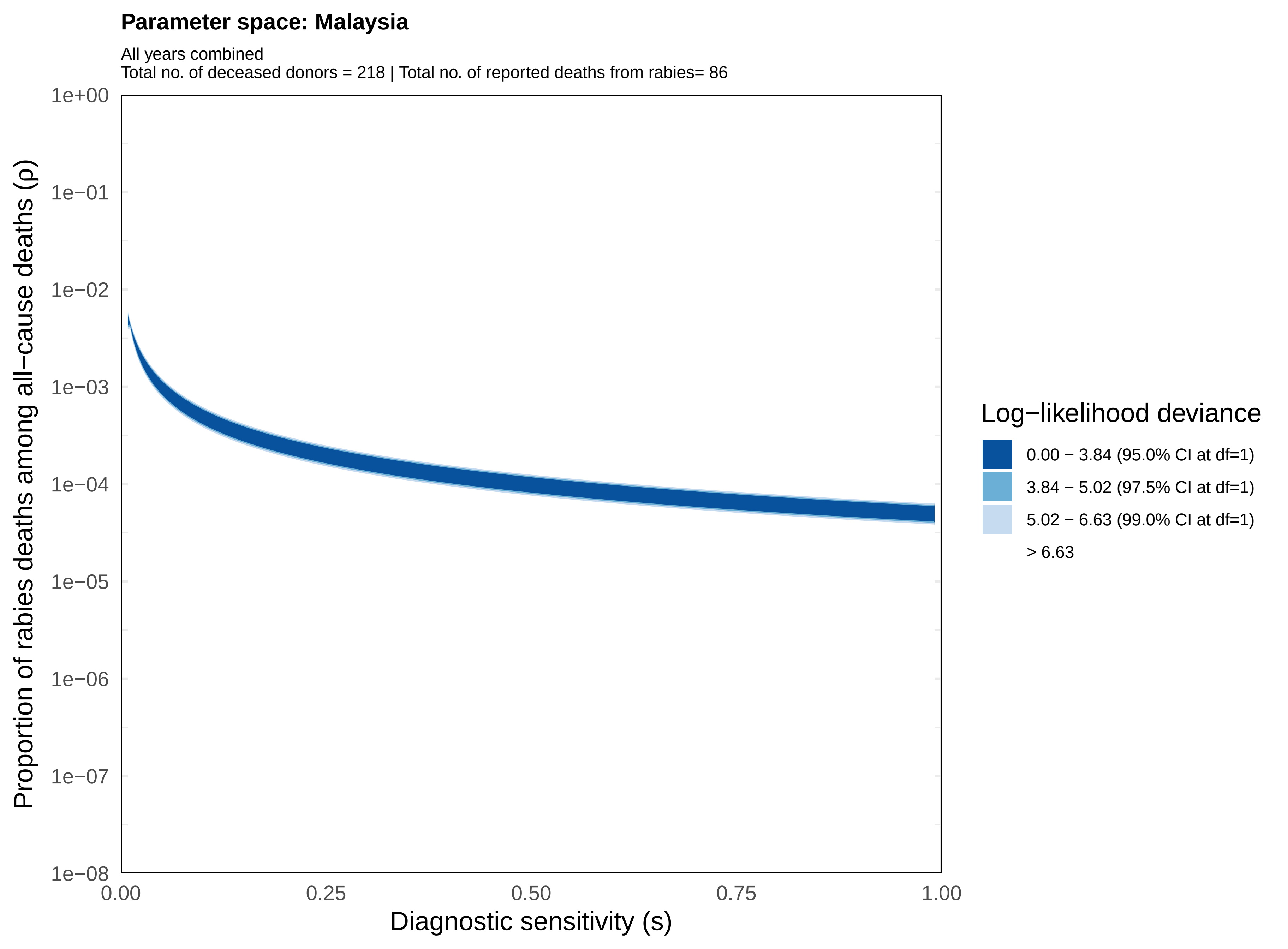

Parameter space: Malaysia
All years combined
Total no. of deceased donors = 218 | Total no. of reported deaths from rabies= 86
1e+00
1e−01
1e−02
1e−03
1e−04
1e−05
1e−06
1e−07
1e−08
Proportion of rabies deaths among all−cause deaths (ρ)
0.00
0.25
0.50
0.75
1.00
Diagnostic sensitivity (s)
Log−likelihood deviance
0.00 − 3.84 (95.0% CI at df=1)
3.84 − 5.02 (97.5% CI at df=1)
5.02 − 6.63 (99.0% CI at df=1)
> 6.63

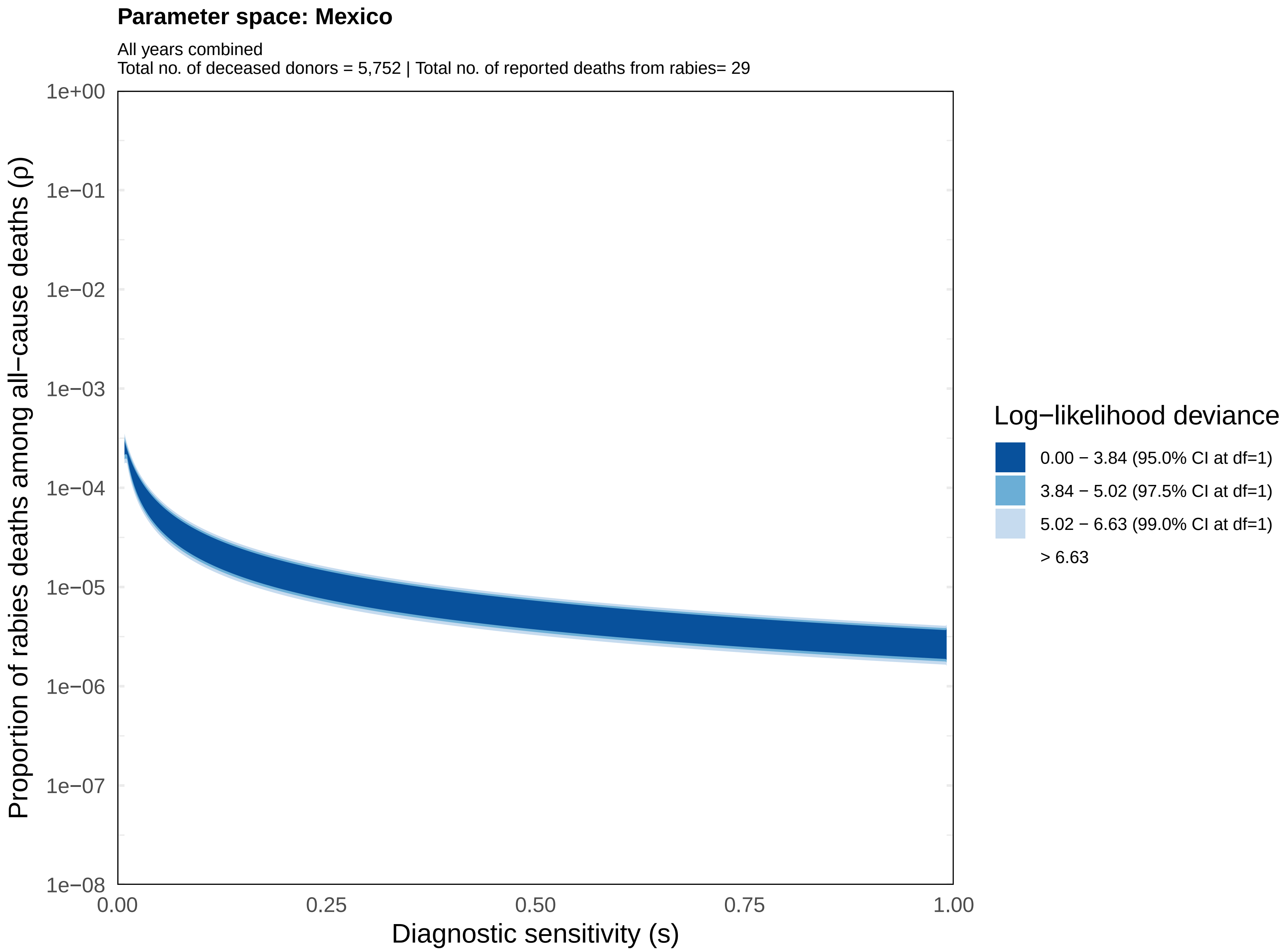

Parameter space: Mexico
All years combined
Total no. of deceased donors = 5,752 | Total no. of reported deaths from rabies= 29
Proportion of rabies deaths among all−cause deaths (ρ)
1e+00
1e−01
1e−02
1e−03
1e−04
1e−05
1e−06
1e−07
1e−08
0.00
0.25
0.50
0.75
1.00
Diagnostic sensitivity (s)
Log−likelihood deviance
0.00 − 3.84 (95.0% CI at df=1)
3.84 − 5.02 (97.5% CI at df=1)
5.02 − 6.63 (99.0% CI at df=1)
> 6.63

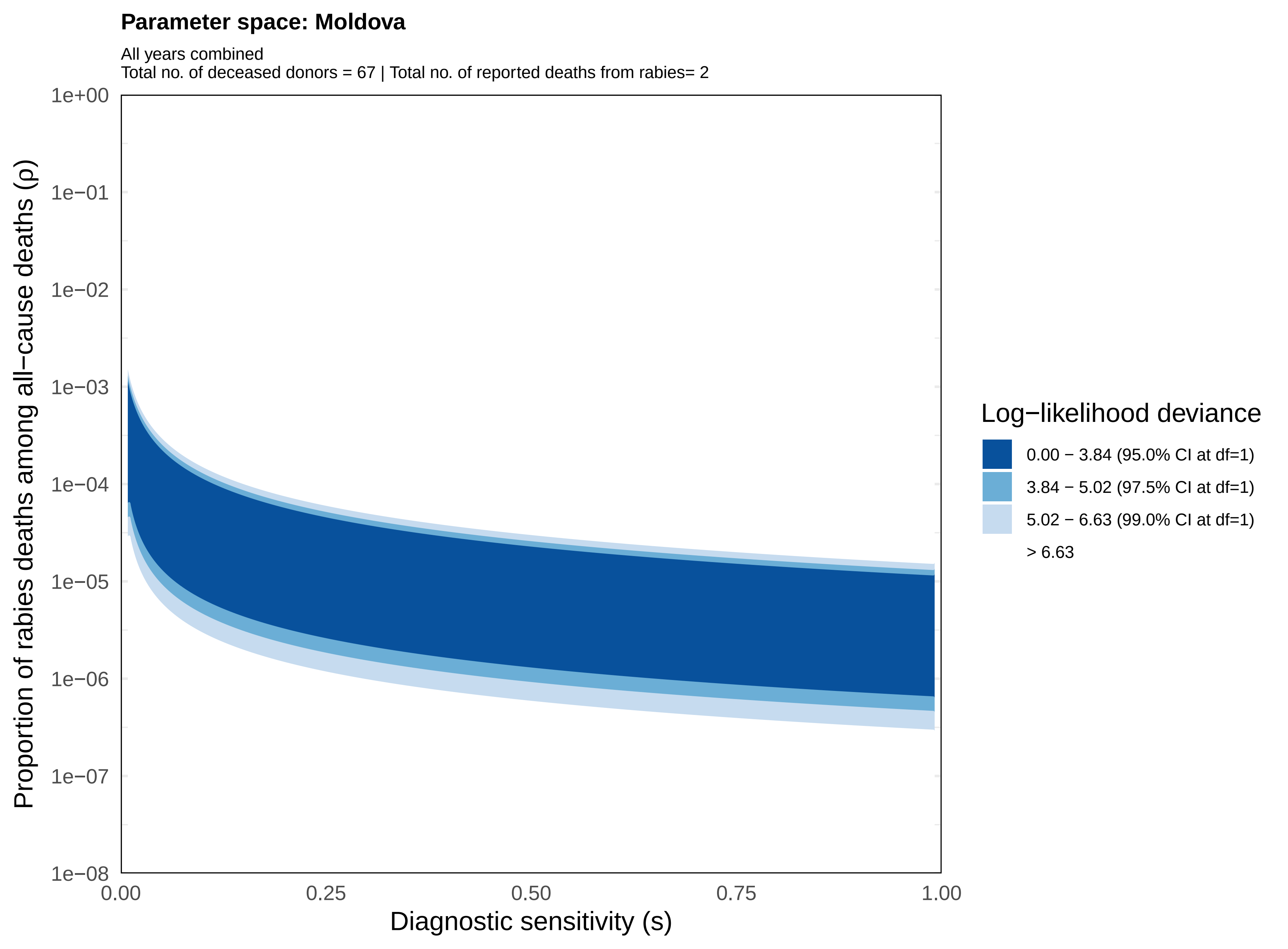

Parameter space: Moldova
All years combined
Total no. of deceased donors = 67 | Total no. of reported deaths from rabies= 2
Proportion of rabies deaths among all−cause deaths (ρ)
1e+00
1e−01
1e−02
1e−03
1e−04
1e−05
1e−06
1e−07
1e−08
0.00
0.25
0.50
0.75
1.00
Diagnostic sensitivity (s)
Log−likelihood deviance
0.00 − 3.84 (95.0% CI at df=1)
3.84 − 5.02 (97.5% CI at df=1)
5.02 − 6.63 (99.0% CI at df=1)
> 6.63

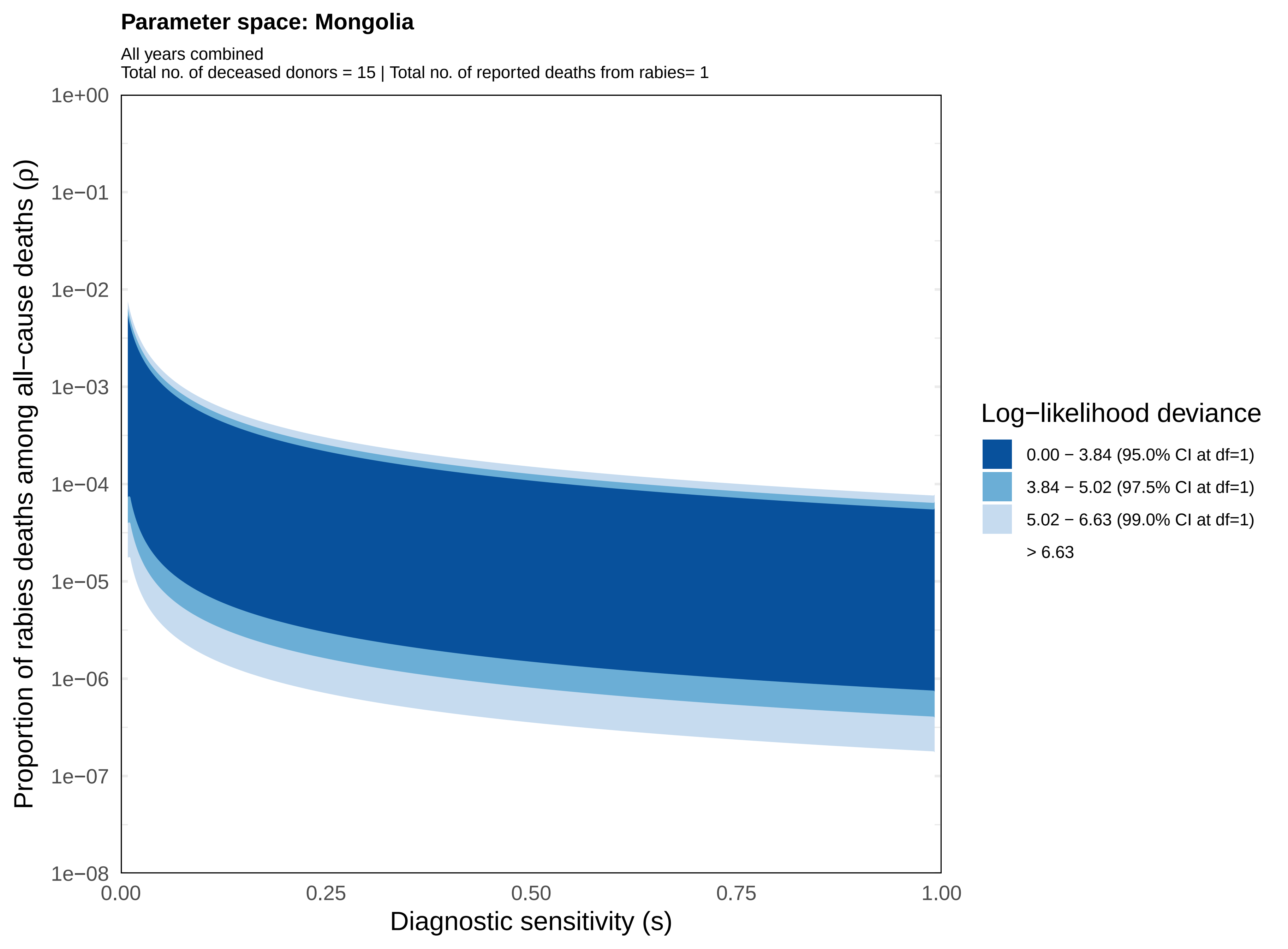

Parameter space: Mongolia
All years combined
Total no. of deceased donors = 15 | Total no. of reported deaths from rabies= 1
1e+00
1e−01
1e−02
1e−03
1e−04
1e−05
1e−06
1e−07
1e−08
Proportion of rabies deaths among all−cause deaths (ρ)
0.00
0.25
0.50
0.75
1.00
Diagnostic sensitivity (s)
Log−likelihood deviance
0.00 − 3.84 (95.0% CI at df=1)
3.84 − 5.02 (97.5% CI at df=1)
5.02 − 6.63 (99.0% CI at df=1)
> 6.63

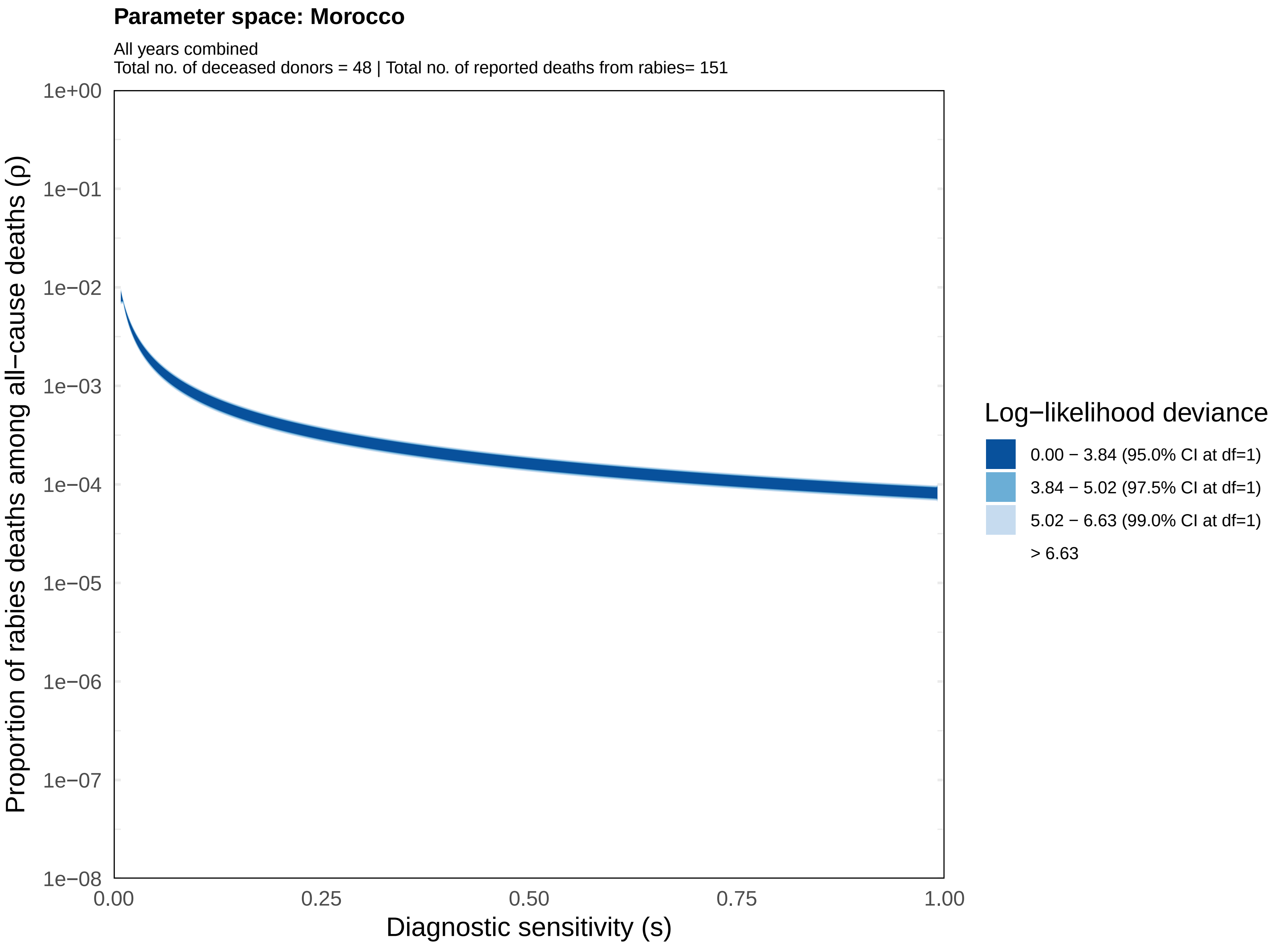
Parameter space: Morocco
All years combined
Total no. of deceased donors = 48 | Total no. of reported deaths from rabies= 151
1e+00
1e−01
1e−02
1e−03
1e−04
1e−05
1e−06
1e−07
1e−08
Proportion of rabies deaths among all−cause deaths (ρ)
0.00
0.25
0.50
0.75
1.00
Diagnostic sensitivity (s)
Log−likelihood deviance
0.00 − 3.84 (95.0% CI at df=1)
3.84 − 5.02 (97.5% CI at df=1)
5.02 − 6.63 (99.0% CI at df=1)
> 6.63

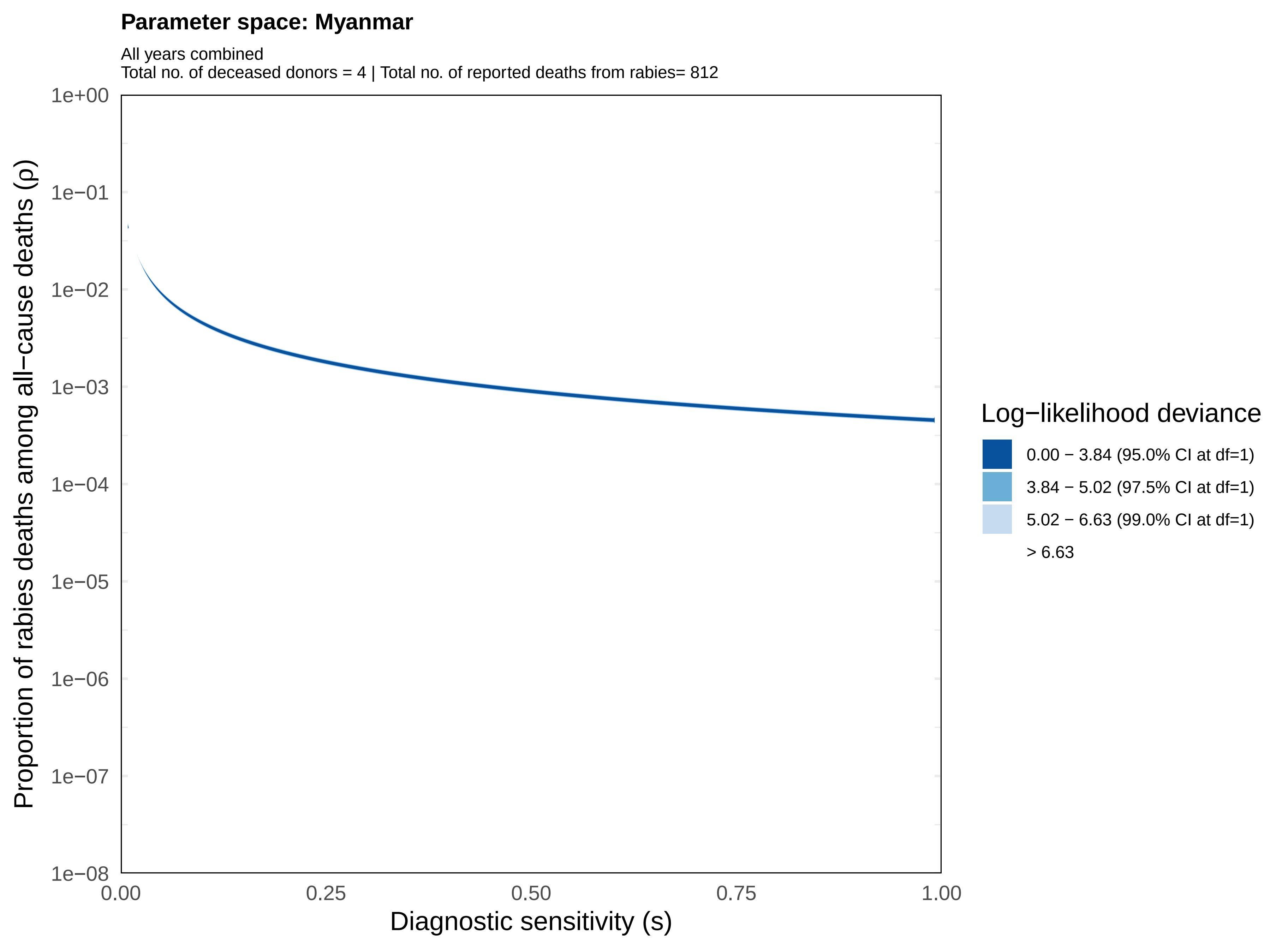

Parameter space: Myanmar
All years combined
Total no. of deceased donors = 4 | Total no. of reported deaths from rabies= 812
1e+00
1e−01
1e−02
1e−03
1e−04
1e−05
1e−06
1e−07
1e−08
Proportion of rabies deaths among all−cause deaths (ρ)
0.00
0.25
0.50
0.75
1.00
Diagnostic sensitivity (s)
Log−likelihood deviance
0.00 − 3.84 (95.0% CI at df=1)
3.84 − 5.02 (97.5% CI at df=1)
5.02 − 6.63 (99.0% CI at df=1)
> 6.63

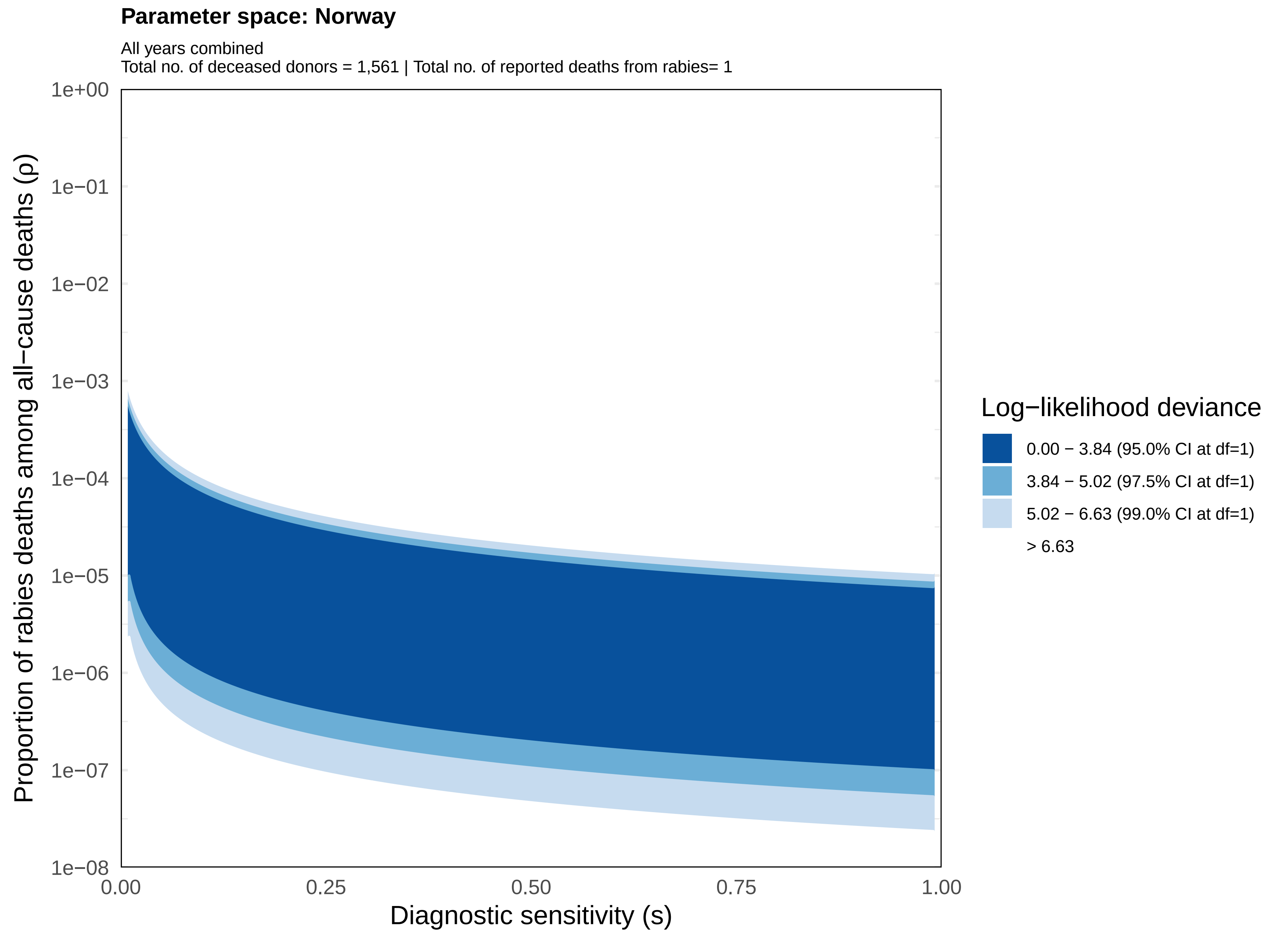
Parameter space: Norway
All years combined
Total no. of deceased donors = 1,561 | Total no. of reported deaths from rabies= 1
1e+00
1e−01
1e−02
1e−03
1e−04
1e−05
1e−06
1e−07
1e−08
Proportion of rabies deaths among all−cause deaths (ρ)
0.00
0.25
0.50
0.75
1.00
Diagnostic sensitivity (s)
Log−likelihood deviance
0.00 − 3.84 (95.0% CI at df=1)
3.84 − 5.02 (97.5% CI at df=1)
5.02 − 6.63 (99.0% CI at df=1)
> 6.63

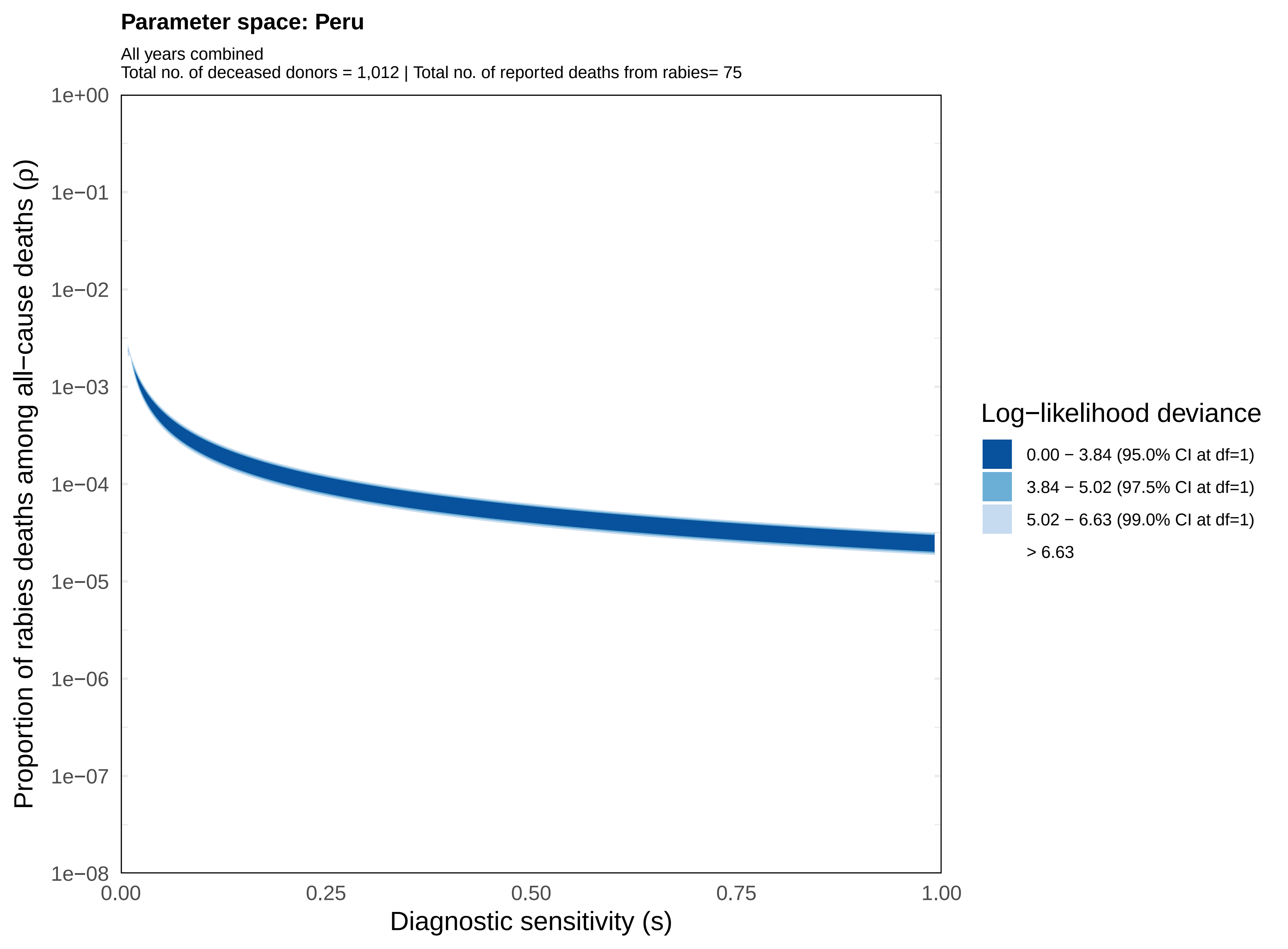

Parameter space: Peru
All years combined
Total no. of deceased donors = 1,012 | Total no. of reported deaths from rabies= 75
Proportion of rabies deaths among all−cause deaths (ρ)
1e+00
1e−01
1e−02
1e−03
1e−04
1e−05
1e−06
1e−07
1e−08
0.00
0.25
0.50
0.75
1.00
Diagnostic sensitivity (s)
Log−likelihood deviance
0.00 − 3.84 (95.0% CI at df=1)
3.84 − 5.02 (97.5% CI at df=1)
5.02 − 6.63 (99.0% CI at df=1)
> 6.63

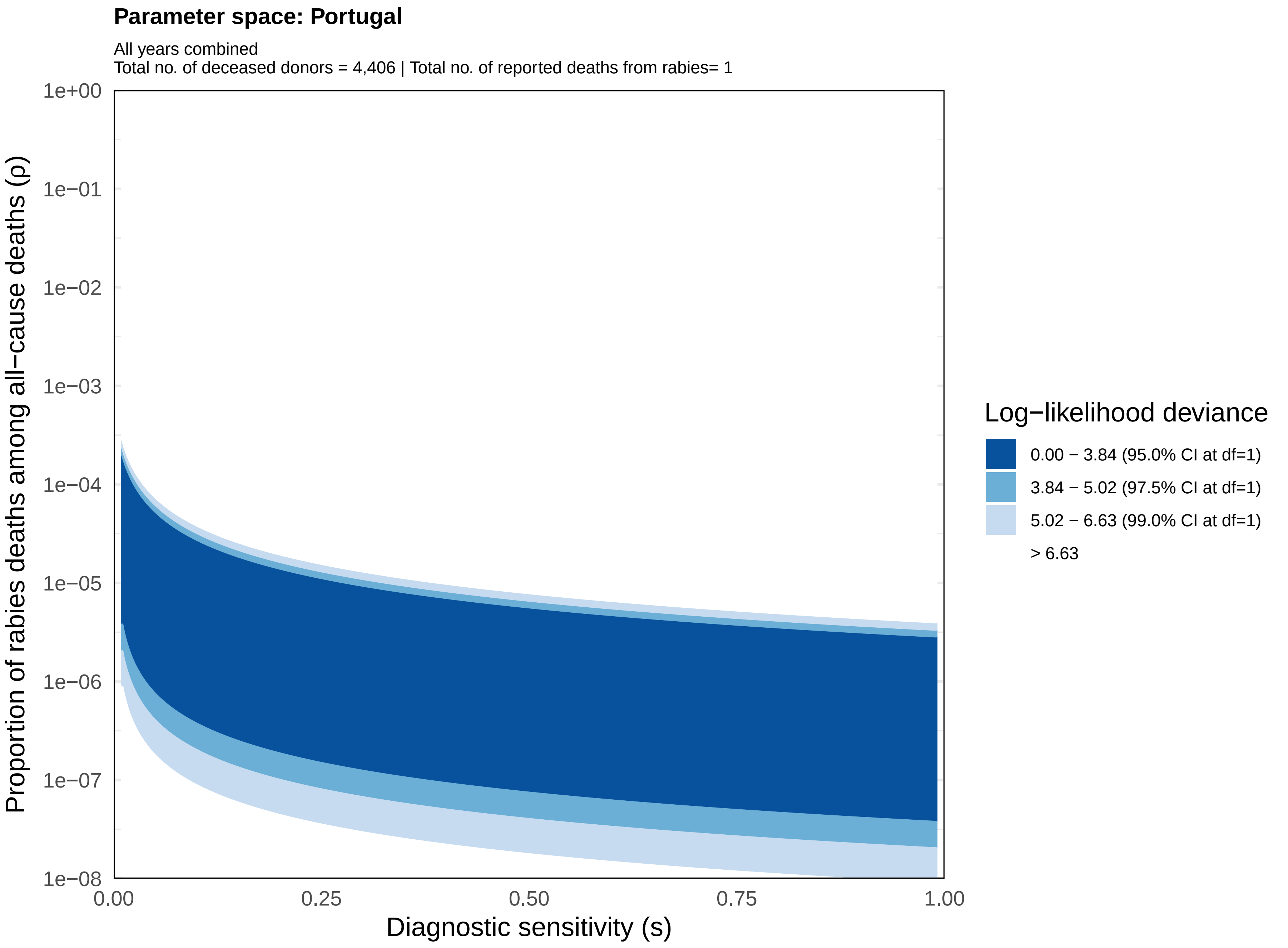

Parameter space: Portugal
All years combined
Total no. of deceased donors = 4,406 | Total no. of reported deaths from rabies= 1
1e+00
1e−01
1e−02
1e−03
1e−04
1e−05
1e−06
1e−07
1e−08
Proportion of rabies deaths among all−cause deaths (ρ)
0.00
0.25
0.50
0.75
1.00
Diagnostic sensitivity (s)
Log−likelihood deviance
0.00 − 3.84 (95.0% CI at df=1)
3.84 − 5.02 (97.5% CI at df=1)
5.02 − 6.63 (99.0% CI at df=1)
> 6.63

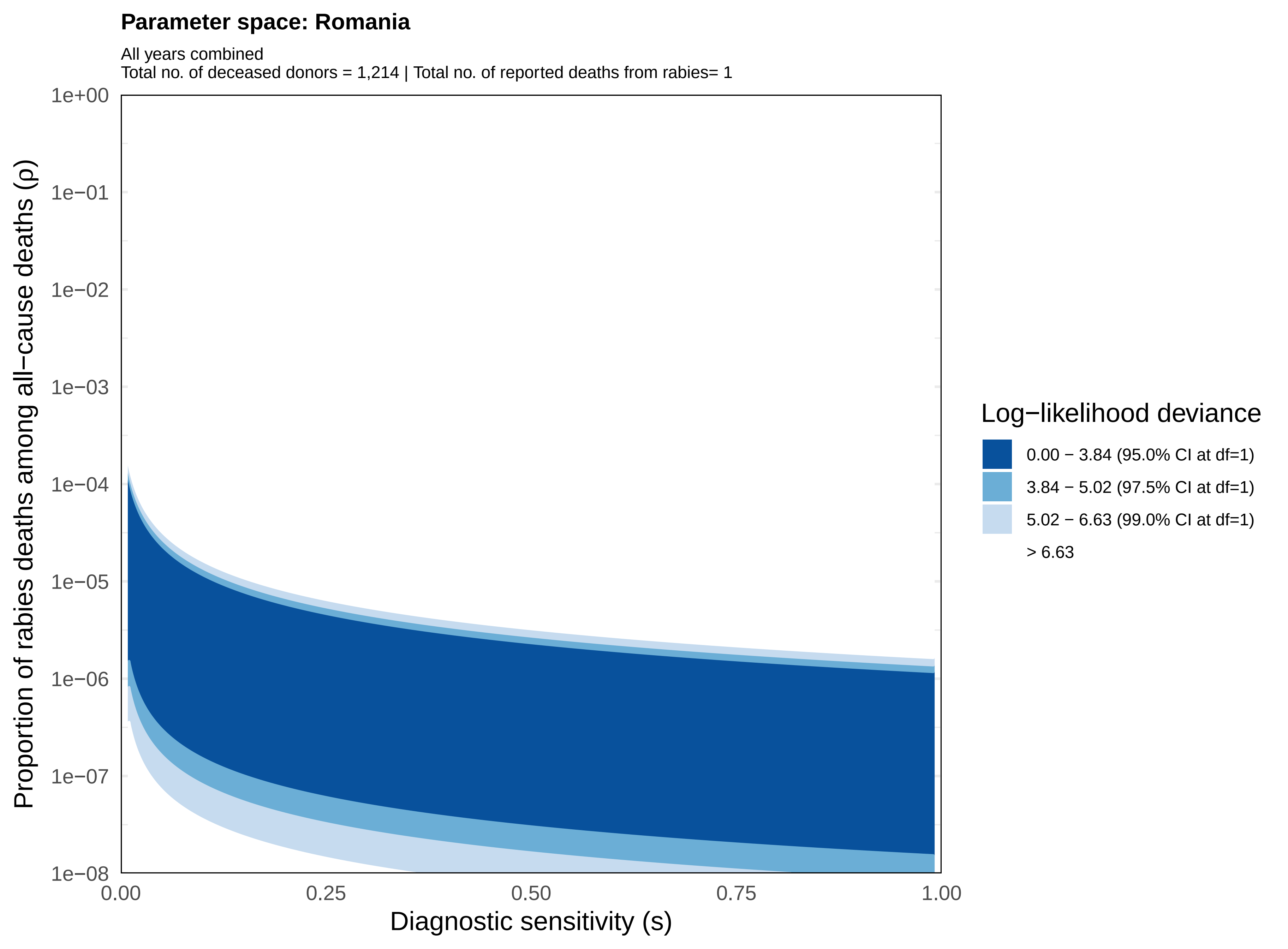
Parameter space: Romania
All years combined
Total no. of deceased donors = 1,214 | Total no. of reported deaths from rabies= 1
Proportion of rabies deaths among all−cause deaths (ρ)
1e+00
1e−01
1e−02
1e−03
1e−04
1e−05
1e−06
1e−07
1e−08
0.00
0.25
0.50
0.75
1.00
Diagnostic sensitivity (s)
Log−likelihood deviance
0.00 − 3.84 (95.0% CI at df=1)
3.84 − 5.02 (97.5% CI at df=1)
5.02 − 6.63 (99.0% CI at df=1)
> 6.63

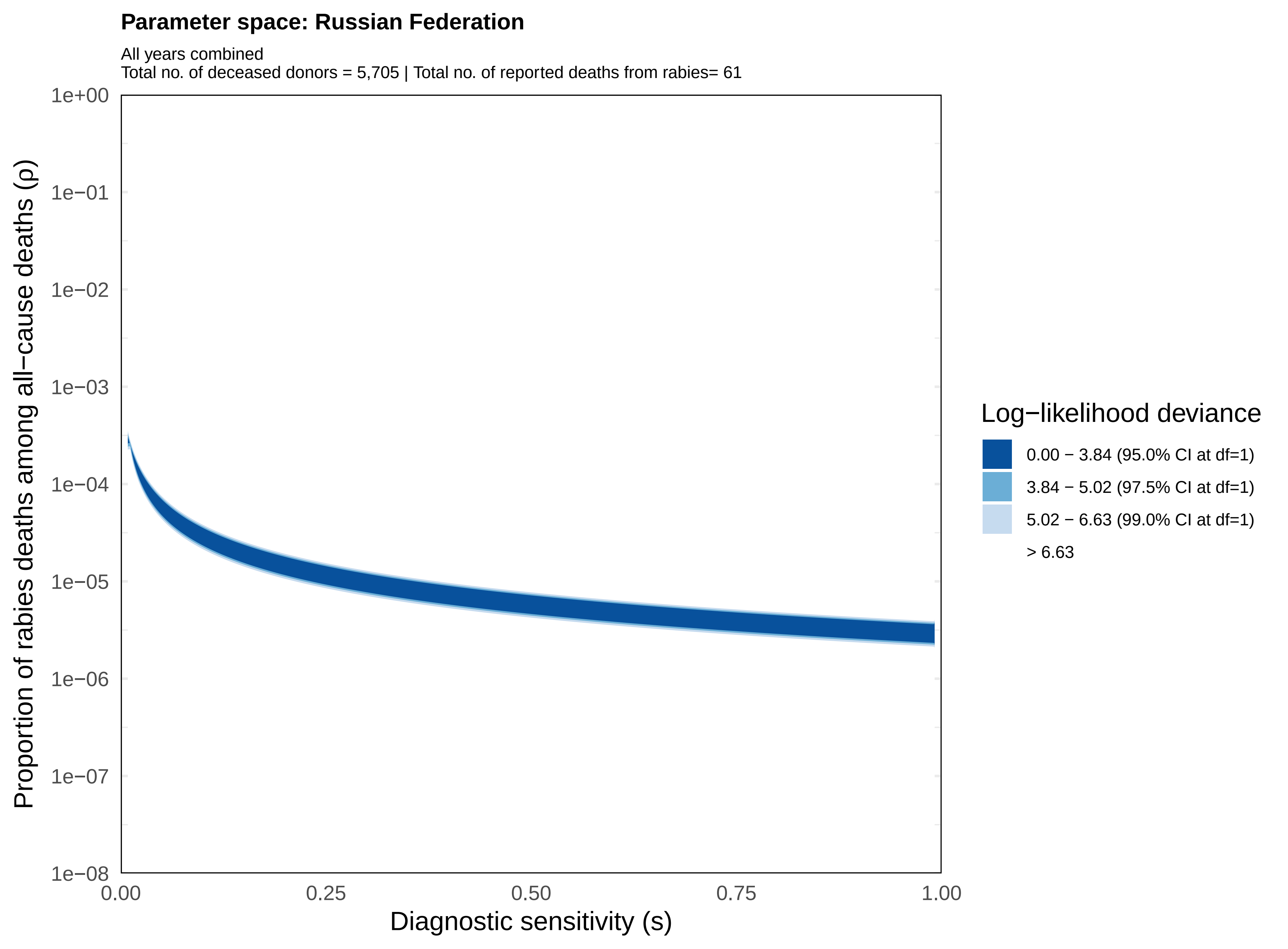

Parameter space: Russian Federation
All years combined
Total no. of deceased donors = 5,705 | Total no. of reported deaths from rabies= 61
Proportion of rabies deaths among all−cause deaths (ρ)
1e+00
1e−01
1e−02
1e−03
1e−04
1e−05
1e−06
1e−07
1e−08
0.00
0.25
0.50
0.75
1.00
Diagnostic sensitivity (s)
Log−likelihood deviance
0.00 − 3.84 (95.0% CI at df=1)
3.84 − 5.02 (97.5% CI at df=1)
5.02 − 6.63 (99.0% CI at df=1)
> 6.63

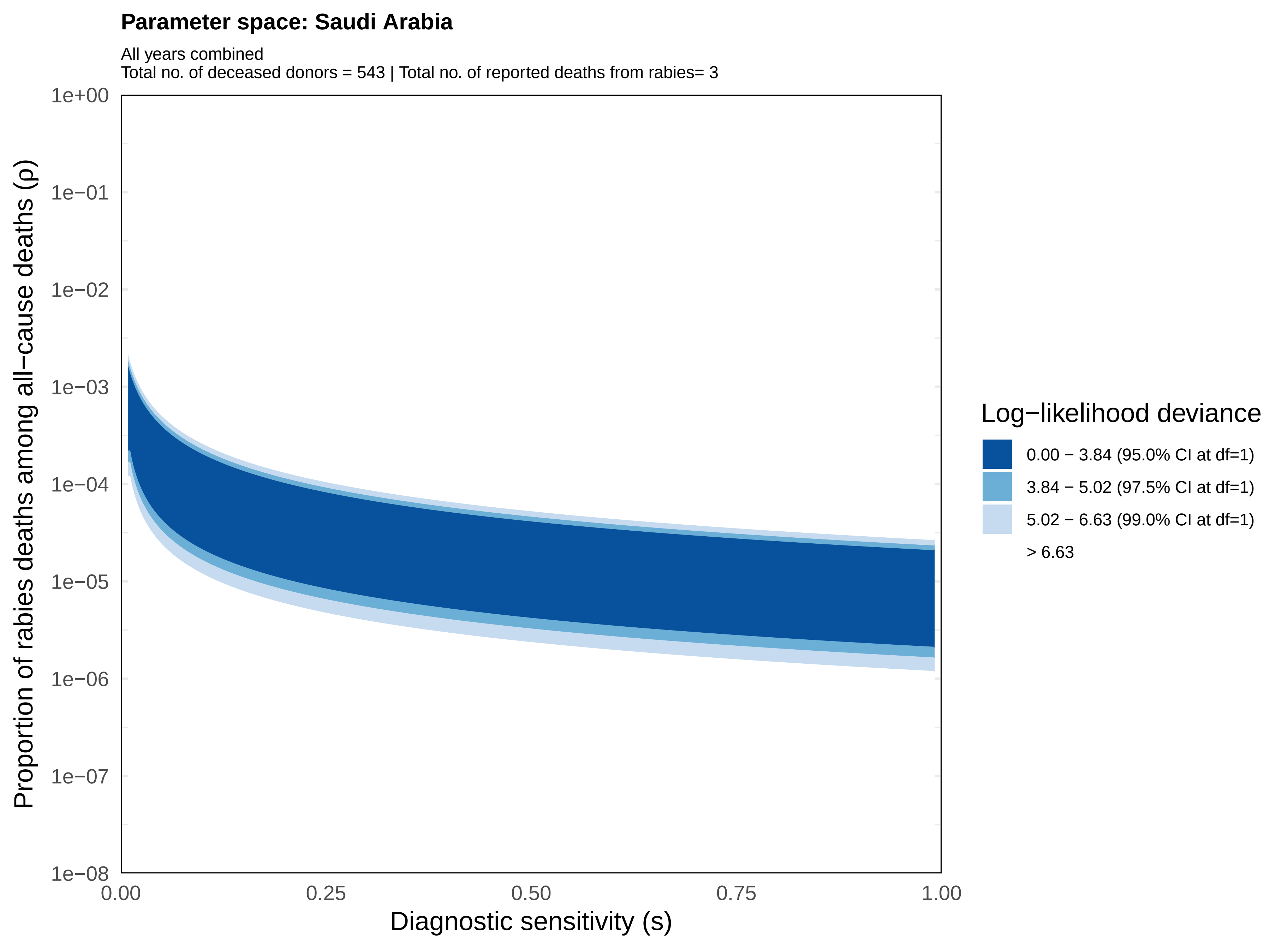

Parameter space: Saudi Arabia
All years combined
Total no. of deceased donors = 543 | Total no. of reported deaths from rabies= 3
Proportion of rabies deaths among all−cause deaths (ρ)
1e+00
1e−01
1e−02
1e−03
1e−04
1e−05
1e−06
1e−07
1e−08
0.00
0.25
0.50
0.75
1.00
Diagnostic sensitivity (s)
Log−likelihood deviance
0.00 − 3.84 (95.0% CI at df=1)
3.84 − 5.02 (97.5% CI at df=1)
5.02 − 6.63 (99.0% CI at df=1)
> 6.63

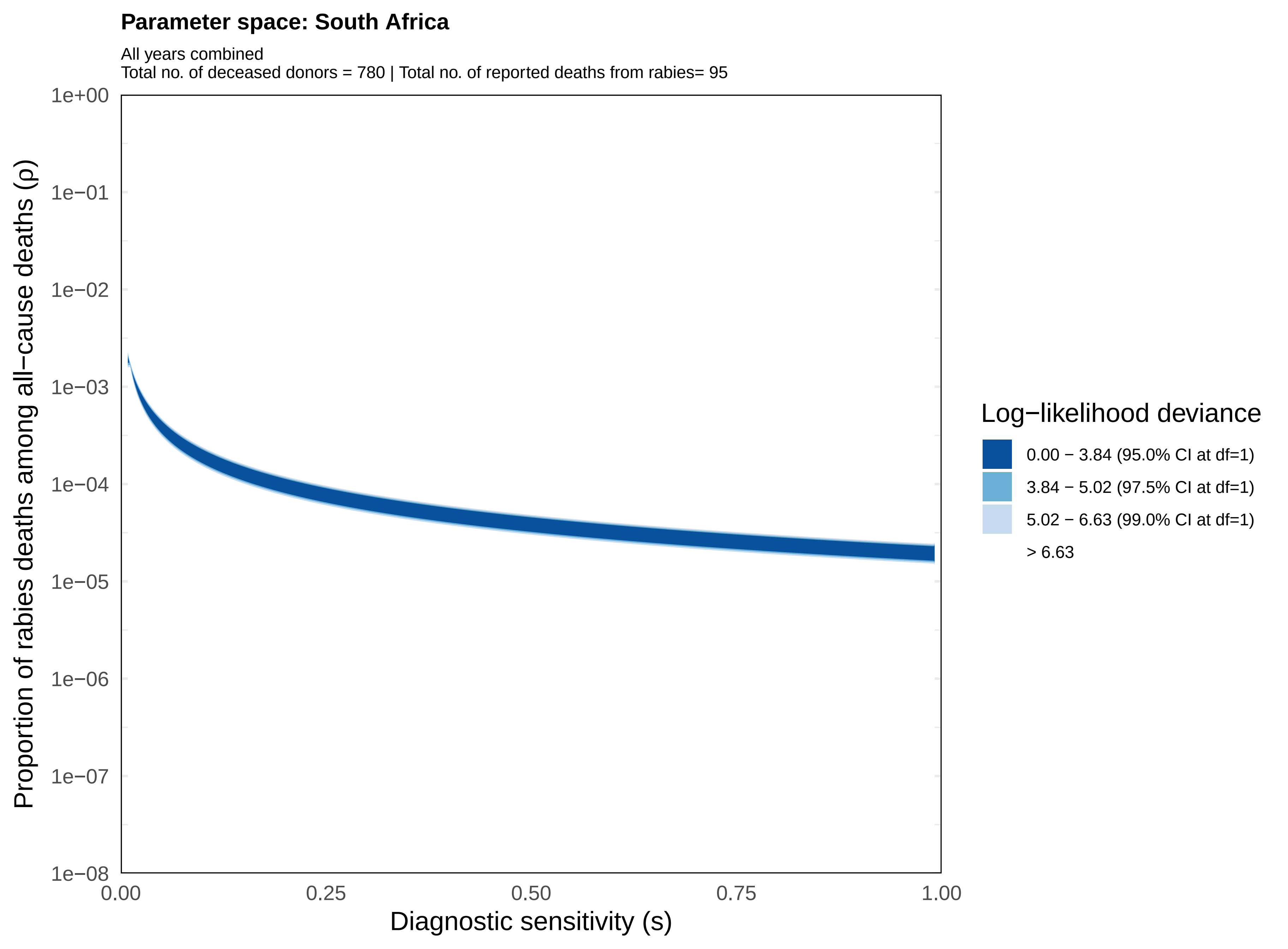

Parameter space: South Africa
All years combined
Total no. of deceased donors = 780 | Total no. of reported deaths from rabies= 95
1e+00
1e−01
1e−02
1e−03
1e−04
1e−05
1e−06
1e−07
1e−08
Proportion of rabies deaths among all−cause deaths (ρ)
0.00
0.25
0.50
0.75
1.00
Diagnostic sensitivity (s)
Log−likelihood deviance
0.00 − 3.84 (95.0% CI at df=1)
3.84 − 5.02 (97.5% CI at df=1)
5.02 − 6.63 (99.0% CI at df=1)
> 6.63

Parameter space: Spain

All years combined
Total no. of deceased donors = 26,969 | Total no. of reported deaths from rabies= 2

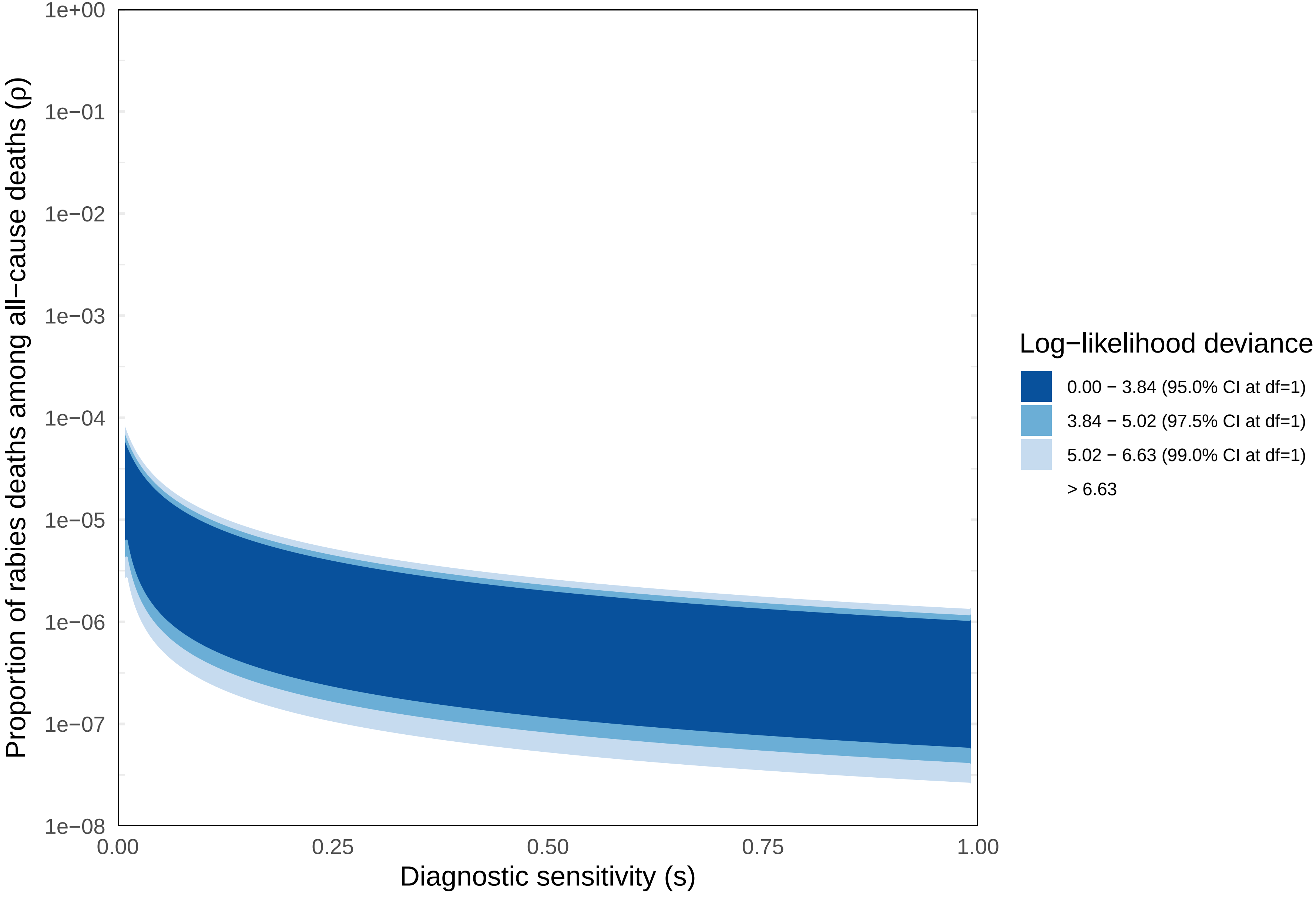

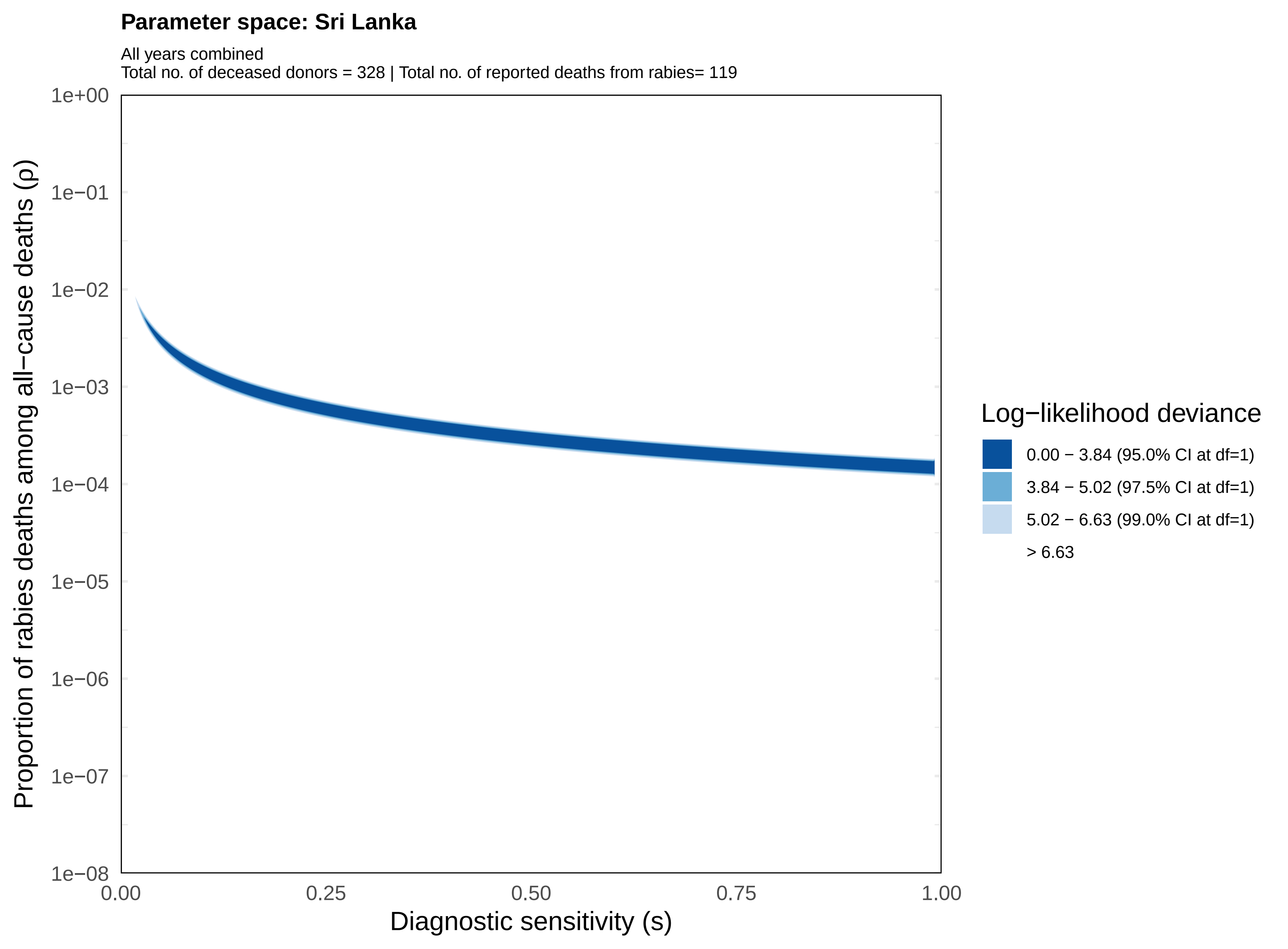
Parameter space: Sri Lanka
All years combined
Total no. of deceased donors = 328 | Total no. of reported deaths from rabies= 119
1e+00
1e−01
1e−02
1e−03
1e−04
1e−05
1e−06
1e−07
1e−08
Proportion of rabies deaths among all−cause deaths (ρ)
0.00
0.25
0.50
0.75
1.00
Diagnostic sensitivity (s)
Log−likelihood deviance
0.00 − 3.84 (95.0% CI at df=1)
3.84 − 5.02 (97.5% CI at df=1)
5.02 − 6.63 (99.0% CI at df=1)
> 6.63

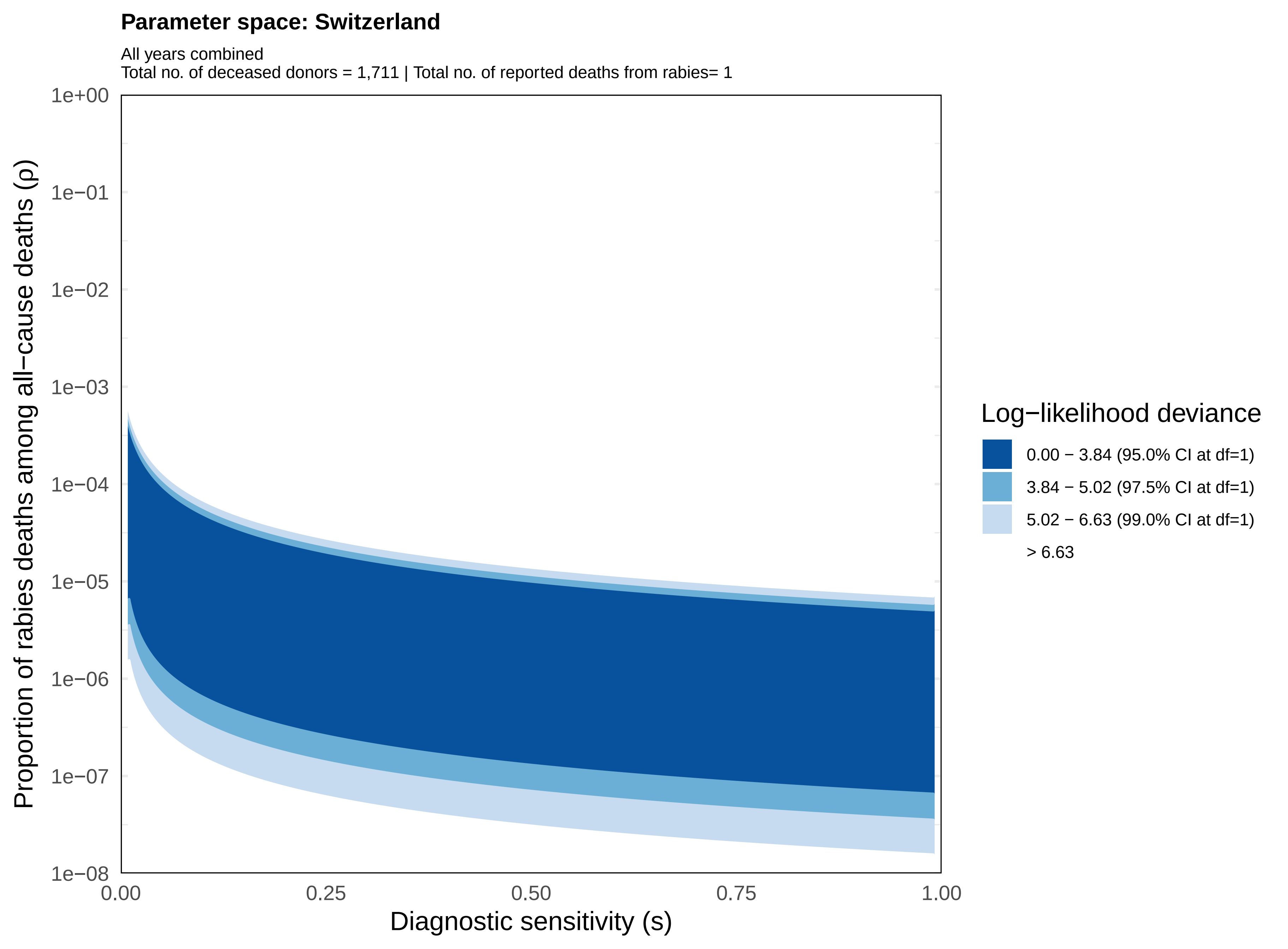

Parameter space: Switzerland
All years combined
Total no. of deceased donors = 1,711 | Total no. of reported deaths from rabies= 1
Proportion of rabies deaths among all−cause deaths (ρ)
1e+00
1e−01
1e−02
1e−03
1e−04
1e−05
1e−06
1e−07
1e−08
Diagnostic sensitivity (s)
0.00
0.25
0.50
0.75
1.00
Log−likelihood deviance
0.00 − 3.84 (95.0% CI at df=1)
3.84 − 5.02 (97.5% CI at df=1)
5.02 − 6.63 (99.0% CI at df=1)
> 6.63

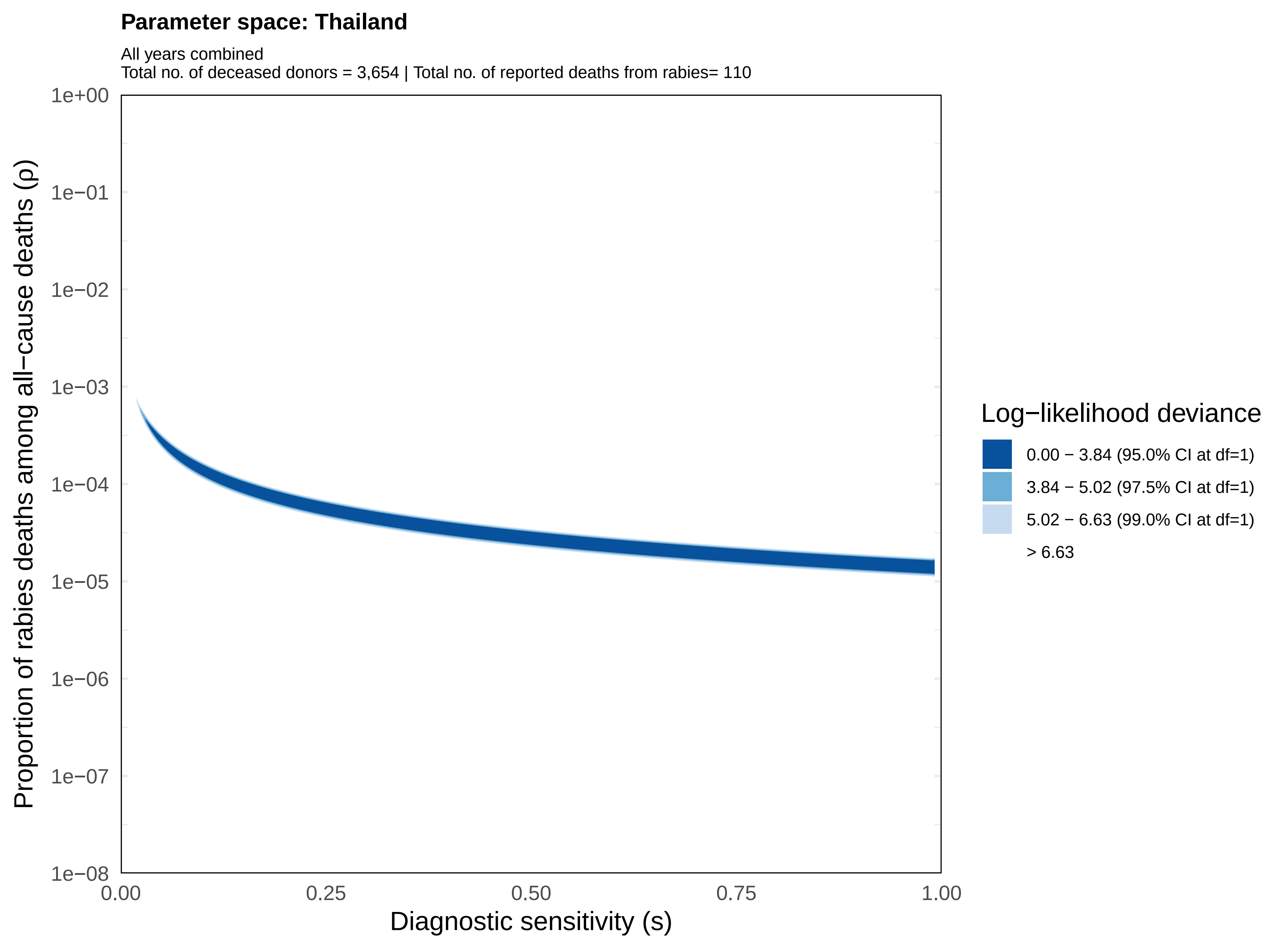
Parameter space: Thailand
All years combined
Total no. of deceased donors = 3,654 | Total no. of reported deaths from rabies= 110
Proportion of rabies deaths among all−cause deaths (ρ)
1e+00
1e−01
1e−02
1e−03
1e−04
1e−05
1e−06
1e−07
1e−08
0.00
0.25
0.50
0.75
1.00
Diagnostic sensitivity (s)
Log−likelihood deviance
0.00 − 3.84 (95.0% CI at df=1)
3.84 − 5.02 (97.5% CI at df=1)
5.02 − 6.63 (99.0% CI at df=1)
> 6.63

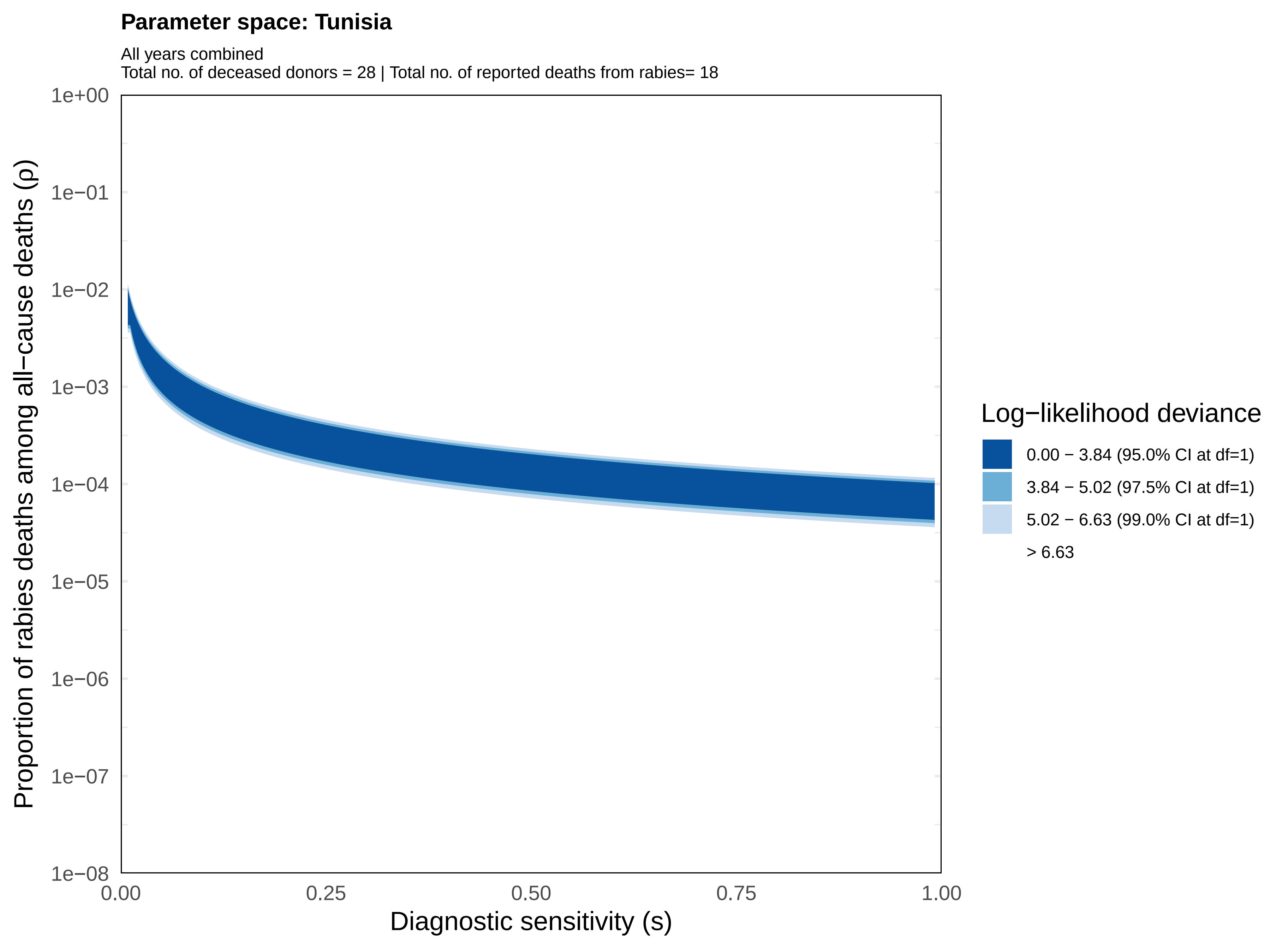

Parameter space: Tunisia
All years combined
Total no. of deceased donors = 28 | Total no. of reported deaths from rabies= 18
1e+00
1e−01
1e−02
1e−03
1e−04
1e−05
1e−06
1e−07
1e−08
Proportion of rabies deaths among all−cause deaths (ρ)
0.00
0.25
0.50
0.75
1.00
Diagnostic sensitivity (s)
Log−likelihood deviance
0.00 − 3.84 (95.0% CI at df=1)
3.84 − 5.02 (97.5% CI at df=1)
5.02 − 6.63 (99.0% CI at df=1)
> 6.63

Parameter space: Ukraine

All years combined
Total no. of deceased donors = 475 | Total no. of reported deaths from rabies= 5

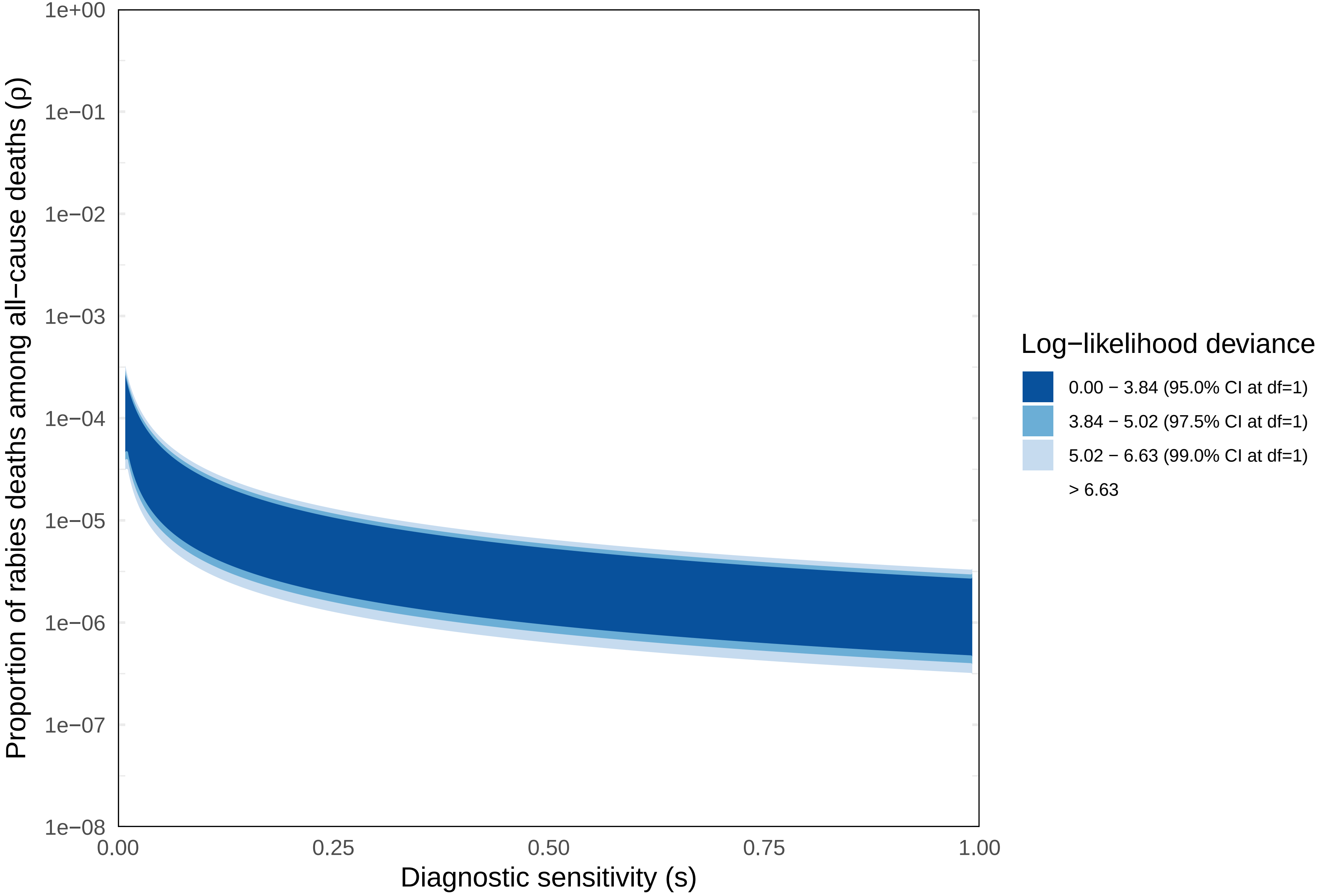

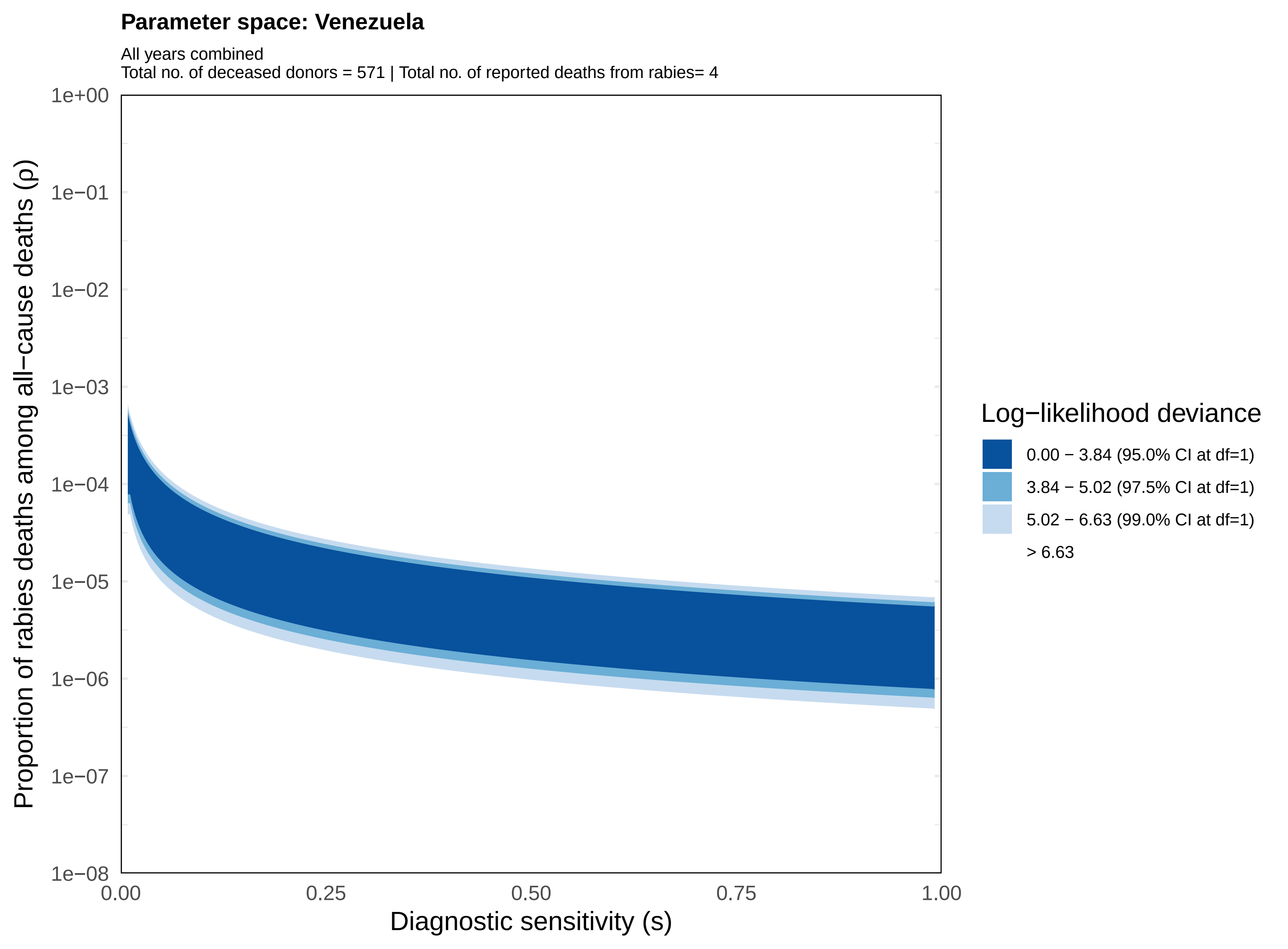
Parameter space: Venezuela
All years combined
Total no. of deceased donors = 571 | Total no. of reported deaths from rabies= 4
1e+00
1e−01
1e−02
1e−03
1e−04
1e−05
1e−06
1e−07
1e−08
Proportion of rabies deaths among all−cause deaths (ρ)
0.00
0.25
0.50
0.75
1.00
Diagnostic sensitivity (s)
Log−likelihood deviance
0.00 − 3.84 (95.0% CI at df=1)
3.84 − 5.02 (97.5% CI at df=1)
5.02 − 6.63 (99.0% CI at df=1)
> 6.63

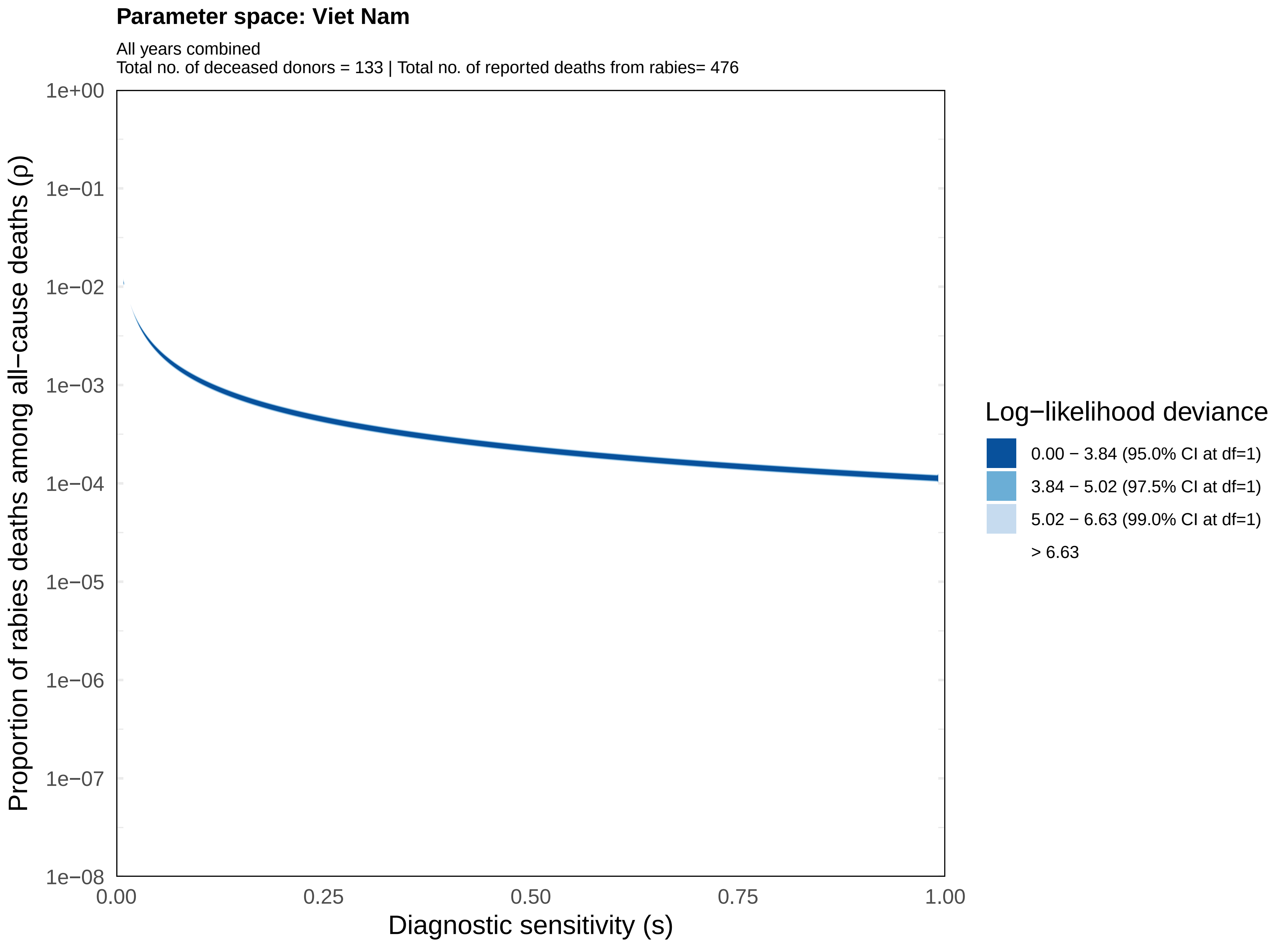
Parameter space: Viet Nam
All years combined
Total no. of deceased donors = 133 | Total no. of reported deaths from rabies= 476
Proportion of rabies deaths among all−cause deaths (ρ)
1e+00
1e−01
1e−02
1e−03
1e−04
1e−05
1e−06
1e−07
1e−08
0.00
0.25
0.50
0.75
1.00
Diagnostic sensitivity (s)
Log−likelihood deviance
0.00 − 3.84 (95.0% CI at df=1)
3.84 − 5.02 (97.5% CI at df=1)
5.02 − 6.63 (99.0% CI at df=1)
> 6.63
